# Statistical modeling of methylene blue degradation by yeast-bacteria consortium; optimization via agro-industrial waste, immobilization and application in real effluents

**DOI:** 10.1186/s12934-021-01730-z

**Published:** 2021-12-30

**Authors:** Marwa Eltarahony, Esmail El-Fakharany, Marwa Abu-Serie, Marwa ElKady, Amany Ibrahim

**Affiliations:** 1 Environmental Biotechnology Department, Genetic Engineering and Biotechnology Research Institute (GEBRI), City of Scientific Research and Technological Applications (SRTA-City), New Borg El-Arab, Alexandria, 21934 Egypt; 2 Protein Research Department, Genetic Engineering and Biotechnology Research Institute (GEBRI), City of Scientific Research and Technological Applications (SRTA-City), New Borg EL-Arab, Alexandria, 21934 Egypt; 3 Medical Biotechnology Department, Genetic Engineering and Biotechnology Research Institute (GEBRI), City of Scientific Research and Technological Applications (SRTA-City), New Borg El-Arab, Alexandria, 21934 Egypt; 4 Chemical and Petrochemical Engineering Department, Egypt-Japan University for Science and Technology, New Borg El-Arab, Alexandria, Egypt; 5 Fabrication Technology Researches Department, Advanced Technology and New Materials Research Institute, City of Scientific Research and Technological Applications (SRTA-City), New Borg EL-Arab, Alexandria, 21934 Egypt; 6 Botany Department, Faculty of Women for Arts, Science and Education, Ain Shams University, Cairo, Egypt; 7 Department of Biology, College of Science, Taif University, P.O. Box 11099, Taif, 21944 Saudi Arabia

**Keywords:** Azo dye, Methylene blue, *Rhodotorula sp.*, Detoxification, Response surface methodology, Plackett–Burman design, Wastewater treatment, Cytotoxicity, Bioaugmentation, Industrial effluents

## Abstract

The progress in industrialization everyday life has led to the continuous entry of several anthropogenic compounds, including dyes, into surrounding ecosystem causing arduous concerns for human health and biosphere. Therefore, microbial degradation of dyes is considered an eco-efficient and cost-competitive alternative to physicochemical approaches. These degradative biosystems mainly depend on the utilization of nutritive co-substrates such as yeast extract peptone in conjunction with glucose. Herein, a synergestic interaction between strains of mixed-culture consortium consisting of *Rhodotorula sp., Raoultella planticola;* and *Staphylococcus xylosus* was recruited in methylene blue (MB) degradation using agro-industrial waste as an economic and nutritive co-substrate. Via statistical means such as Plackett–Burman design and central composite design, the impact of significant nutritional parameters on MB degradation was screened and optimized. Predictive modeling denoted that complete degradation of MB was achieved within 72 h at MB (200 mg/L), NaNO_3_ (0.525 gm/L)_,_ molasses (385 μL/L), pH (7.5) and inoculum size (18%). Assessment of degradative enzymes revealed that intracellular NADH-reductase and DCIP-reductase were key enzymes controlling degradation process by 104.52 ± 1.75 and 274.04 ± 3.37 IU/min/mg protein after 72 h of incubation. In addition, azoreductase, tyrosinase, laccase, nitrate reductase, MnP and LiP also contributed significantly to MB degradation process. Physicochemical monitoring analysis, namely UV−Visible spectrophotometry and FTIR of MB before treatment and degradation byproducts indicated deterioration of azo bond and demethylation. Moreover, the non-toxic nature of degradation byproducts was confirmed by phytotoxicity and cytotoxicity assays. *Chlorella vulgaris* retained its photosynthetic capability (˃ 85%) as estimated from Chlorophyll-a/b contents compared to ˃ 30% of MB-solution. However, the viability of Wi-38 and Vero cells was estimated to be 90.67% and 99.67%, respectively, upon exposure to MB-metabolites. Furthermore, an eminent employment of consortium either freely-suspended or immobilized in plain distilled water and optimized slurry in a bioaugmentation process was implemented to treat MB in artificially-contaminated municipal wastewater and industrial effluent. The results showed a corporative interaction between the consortium examined and co-existing microbiota; reflecting its compatibility and adaptability with different microbial niches in different effluents with various physicochemical contents.

## Introduction

Despite the recent economic prosperity generated by the progress in industrialization and urbanization, both are considered a major reason of environmental pollution. Several hazardous xenobiotic compounds are introduced into the biosphere and water resources from various manufactures, synthetic dyes are among. More than 100,000 chemically diverse dyestuffs are commercially available. Based on industrial application method, dyes are classified into acid, basic, reactive, vat, direct and dispersed [1–4]. However, they are categorized into azo, indigo, anthraquinone, phthalocyanine, nitroso and nitro, etc., according to their chemical nature [3, 5]. As reported by [6, 7], azo dyes account for more than 70% of commercially used organic dyes by the dint of their ease in synthesis, higher water solubility and higher stability under different conditions such as temperature, light, detergent and microbial deterioration. Their chemical structure could be described as an aromatic system conjugated to one or more of chromophore azo (–N=N–) groups and an auxochrome sulfonic (SO^−3^) groups associated with hydroxyl, methyl, chloro, triazine amine and nitro [1, 3]. Among them, methylene blue (MB), which is called (3,7-is(Dimethylamino)-phenothiazin-5-iumchloride) according to IUPAC nomenclature. It is aromatic heterocyclic cationic dye that has been primarily utilized as a medication for methemoglobinemia and urinary tract infections as cited in [8]. Besides, it is used as tissue stain in surgical process, antiseptics and pH-indicators in biological laboratories. Additionally, it is utilized in cosmetics, photography, pharmaceutical, food, cardboard, paper, leather and painting industries. Nowadays, it is employed in dying cotton, wood, silk, hair, nylon, rayon, and other textile fibers; besides, colouring of plastics, oils, gasoline and waxes [9–12].

The inefficient dyeing process, in particular the fixation step, causes 10–70% of the amount used being discharged into the aquatic ecosystem as effluents [6–8, 13]. In fact, the concentration of unfixed dyes in textile effluents is estimated to be 10–200 mg/L [7, 14]. However, their presence in negligible concentrations in the range of 10–50 mg/L is considered a major environmental and public health concern [7, 15]. At such concentrations, the water transparency is adversely influenced by clearly visible dye, which certainly exerts several impacts such as diminishing light penetration, blocking photosynthesis of phytoplankton and algae, altering pH, reducing dissolved oxygen (DO), elevating the biological oxygen demand (BOD) and chemical oxygen demand (COD), which ultimately disrupt the quality of aquatic ecosystem. Moreover, the presence of azo dyes or their aromatic by-products in water causes shortness of breath, vomiting, diarrhea, sweating, allergy and hypopigmentation. Notably, some carcinogenic, mutagenic and neurotoxic effects of them have also been reported [7, 15, 16]. Therefore, to reduce these environmental pollution and human health risks, the environmental legislation has enforced dyestuff manufacturers to treat effluents contaminated with dyes prior to their discharge into open streams [7].

Numerous and various strategies of physicochemical approaches are available to address the treatment process, such as adsorption, flocculation, membrane filtration, sedimentation, coagulation, photocatalysis, ozonation and advance oxidation process (AOP) [17]. However, these methods encountered several drawbacks such as generation of significant amounts of sludge along with risky by-products, which are deemed as secondary pollution, incomplete removal, unfeasible economically, high energy/reagent requirements, complicated technical operations, high cost of maintenance and post-treatment problems such as fouling. Relatively, the employment of biological system for treatment process intensified recently due to eco-friendly nature, inexpensive, nontoxic and less-sludge/toxic by-products output as cited in [18]. This bioremediation process depends on the utilization of plants, microbes (e.g., bacteria, actinomycetes, yeasts, molds and algae) and/or their enzymes to fulfil the task of degradation and detoxification [19]. Different microbial forms (live, dead biomass) under various conditions (aerobic, anaerobic, microaerophilic) combined or sequential were reported in this regard [18]. In fact, most studies focused on bacterial degradation due to the faster growth rate, pervasiveness and susceptibility to physiological and genetic manipulation as stated by [17, 20]. Interestingly, [21] showed a promising removal of MB by *Bacillus albus* isolated form textile sludge sample. Besides, [22] declared that *Acinetobacter pittii* screened from dye house effluent exhibited bioremediation potency of MB reached 73% within 5 days. In the same sense, [23] recorded that *Pseudomonas aeruginosa* degraded 82.25% of MB (50 mg/L) within 24 h. of incubation.

Nonetheless, [24] studied the biodegradation of azo dye by *Galactomyces sp*. and *Aspergillus terreus*, respectively. Additionally, [25] utilized *Phanerochaete chrysosporium* to degrade MB in agricultural residues. Moreover, [26] recorded the biotransformation of MB (100 mg/L) in potato dextrose broth by *Daedalea dickinsii* after 14 days.

Interestingly, the application of mixed culture or microbial consortium is considered technically attractive more than monoculture. Where, sole microbial culture would exhibit slower degradation efficiency in comparison to mixed culture. That could be attribute to vulnerable acclimatization of single culture to pollutants, which consequently influences enzymatic activity.

[27]. However, the presence of mixed cultures circumvents this demerit by the syntrophic action of numerous detoxification enzymes provided by mixed bacterial cultures [19, 28]. As described by [17], the incomplete utilization of azo dye by a single microbial culture could results in intermediate metabolic by-product that would be consumed by co-existing strain; eventually complete decomposition without any toxic residues. It is noteworthy that the adaptability and compatibility are the decisive parameters that control this process [17, 23]. Indeed, different microbial groups were applied in their combinatorial forms to remediate organic pollutants, including bacterial–bacterial [17, 29, 30], fungal-fungal [31], algal–algal [32], bacterial–fungal [33], bacterial–algal [34] and fungal–algal [35].

A paramount trait of biodegrading or biomineralizing microbes is a battery of oxidoreductive enzymes. Several researches demonstrated the crucial role of some azoreductases and oxidative enzymes in the detoxification process such as laccase, N-demethylase manganese peroxidases (MnP), veratryl alcohol oxidase, tyrosinase, cellobiose dehydrogenase, lignin peroxidases (LiP) and NADH-dependent reductases. In general, azoreductases and NADH-dependent reductases degrade dye anaerobically; whereases, the others decompose dye aerobically [5]. However, the genetic diversity and metabolic versatility of microbes in mixed culture complement their degradative capacities by corporative effect of enzymatic network [19].

Interestingly, the utilization of statistical design of experiments (DOEs) such as Plackett–Burman design (PBD) and response surface methodology (RSM) in bioremediation process is increasing nowadays [36–38]. Through these chemometric approaches, the optimized condition of multivariable system will be determined. Let alone defining the individual and interaction effect of each independent variable within the studied ranges in cost-effective and less time/effort mean [36, 38]. There is a plethora of scientific researches addressed biodegradability of various azo dyes in rich medium supplemented with complex co-substrates such as glucose, peptone, yeast extract or their combination. However, no study reported decolorization process in the presence of agro-industrial waste, like molasses, as an economic and cheap co-substrate for enhancing degradation performance. Consequently, it serves as alternative mean for complex nutritive ingredients that could be utilized by degradative microbes to gain electrons that would be directed to the reductive breakdown of azo bond. Therefore, the recruitment of such agro-industrial waste seems being a promoting alternative in treatment of anthropogenic pollutant economically [39].

In this context, the current study focused on the utilization of yeast–bacterial co-cultures consortium in MB degradation. The cooperative interactions between yeast and bacteria were optimized by statistical design to achieve enhanced MB-detoxification and degradation. Herein, the optimization process was performed using nutrients-poor medium, supplemented with molasses as a waste liquor co-substrate to be more economic for application in dyestuff containing effluents. To the best of authors acquaintance, no study has addressed the utilization of agro-industrial waste as a co-substrate for dye detoxification by yeast–bacteria association. Thereafter, the oxidoreductive enzymes produced by the designed consortium were assessed by standard assays. Moreover, the biodegradation performance was confirmed by UV–Visible spectra and FTIR analyses. Besides, the environmental impact of the biodegradation metabolites by-products was monitored using acute toxicity assays against algae and human cell lines. Finally, the immobilized consortium, either in optimized slurry or in plain distilled water, was applied in artificially contaminated effluents in a comparative manner, deeming that a scalable solution for biodegradation of azo dyes from industrial effluents in a manner that mimic field conditions.

## Materials and methods

### Screening of MB-detoxifying microorganisms, selection and growth condition.

In the current study, the indoor culture collection of 16S and 18S-rDNA-identified strains (bacteria, actinomycetes, yeast and molds) isolated from various environmental niches and industrial discharges were screened for MB detoxification using plate assay. On Mineral salt basal medium (MSBM) (g/L) (K_2_HPO_4_, 1.27; MgSO_4_.7H_2_O, 0.42 g; NaNO_3_, 0.42 g; NaCl, 2; KH_2_PO_4_, 0.85 g, pH 7.0) accompanied by 100 μL of molasses and 50 mg of MB [40], about 50 μL of microbial inoculum were spotted individually on the surface of the solidified agar plates and incubated at 30 and 25 °C for 72 h for bacteria and fungi, respectively. The preliminary selection of MB-detoxifying microbes was determined by the presence of clearance zone surrounding colonies. According to the diameter of clearance zone and subsequently the degradative capability, the microbial colonies with the largest decolorization areas were chosen.

### Consortium development and compatibility assessment

Three representative strains, i.e., MNR, AM1, and MMT, each categorized to different phyla were utilized for the development of consortium. The microbial cultures were prepared by inoculating a loopful of each microbe separately in Nutrient Broth (NB) medium and incubated in orbital shaker with 150 rpm at 30 °C for 24 h. The microbial cultures were harvested by centrifugation at 10,000*g* for 10 min, and then the pellets were washed twice with sterilized distilled water. Each microbial pellet was adjusted to an OD_600_ of 1.0 and ensuring its performance to detoxify MB in liquid fed-batch state. The mixed-culture consortium was constructed by aseptically combination of equal density of each microbe and its proficiency was also examined in the same way [39]. The antagonism test was performed to confirm the compatibility among consortium strains which would facilitate MB removal process. Briefly, three NB plates were used. After solidification, two wells per each plate were punctured using sterile cork-borer (9 mm). A 50 μL microbial lawn (0.5 McFarland equivalents to 10^8^ CFU/mL) of strains MNR, AM1 and MMT were swapped separately on each NB plate. Each swapped plate was inoculated alternatively with 50 μL of the other two strains in each well. In this way, the antagonism effect of two adapted microbes was tested on the growth of the swabbed one. The plates were incubated as formerly described. The presence of compatibility and absence of antagonism was proven by the absence of clear area around each well [31]. Thus, the obtained consortium was used as an inoculum source for the remaining steps.

### Optimization of MB detoxification conditions

#### Screening for parameters affecting the MB removal via Plackett–Burman design

To optimize cultural and nutritional conditions for MB detoxification with maximum yield, statistical design of experiment (DOE) was employed. All decolorization trials were conducted in 250 mL Erlenmeyer flask containing 100 mL media. DOE was performed through two successive steps, Plackett–Burman statistical design followed by central composite design (CCD).

PBD is identified as a fraction of a two-level factorial design that is dedicated to screen a large number of input parameters (nutritional and incubation conditions) in the fewest number of experimental trials compared to those required by common one-variable-at-a-time (OVAT). Subsequently, the most significant factors affecting the response were determined based on their main effect [41]. The PBD matrix was designed for 11 independent variables in 12 experimental runs (Table 1). Each independent variable was examined at two levels, high ( +) and low (−); where, the number of (–) is equal to (N − 1)/2 and the number of ( +) is equal to (N + 1)/2 in a row. A column contains equal number of (+) and (−) signs. Each trial was performed in triplicates and the mean values of MB degradation represent the response. The main effect of each variable was calculated as the difference between the average of measurements assessed at both high setting (+ 1) and low setting (− 1). PBD is based on the first order model (Eq. 1) [42, 43]:

1 $${\text{Y}} = \beta O + \Sigma \beta {\text{i}}{\rm X}{\text{i}}$$

**Table 1:** PBD matrix of independent variables and their concentration with MB degradation as response

Run order	NaCl	MgSO_4_	KH_2_PO_4_	K_2_HPO_4_	NaNO_3_	MB	Molasses	Inoculum Size (CFU/mL)	Incubation Time (days)	PH	Experimental MB degradation	Predicted MB degradation
	(gm/L)						(μL)				%	
**1**	1	0.63	0.45	1.9	0.21	0.5	300	10^6^	3	5	5.7	5.533
**2**	1	0.21	0.15	1.9	0.63	0.3	300	10^12^	3	5	37.7	37.867
**3**	3	0.21	0.45	0.65	0.21	0.5	100	10^12^	3	5	3.8	3.967
**4**	1	0.63	0.15	0.65	0.21	0.5	300	10^12^	1	9	2.3	2.467
**5**	3	0.63	0.15	1.9	0.63	0.5	100	10^6^	1	5	1.7	1.867
**6**	3	0.21	0.45	1.9	0.21	0.3	300	10^6^	1	5	6.6	6.767
**7**	1	0.63	0.45	0.65	0.63	0.3	100	10^6^	3	9	4.4	4.567
**8**	3	0.63	0.15	1.9	0.21	0.3	100	10^12^	3	9	7.3	7.133
**9**	3	0.63	0.45	0.65	0.63	0.3	300	10^12^	1	5	23.7	23.533
**10**	3	0.21	0.15	0.65	0.63	0.5	300	10^6^	3	9	5.1	4.933
**11**	1	0.21	0.45	1.9	0.63	0.5	100	10^12^	1	9	2.3	2.133
**12**	1	0.21	0.15	0.65	0.21	0.3	100	10^6^	1	5	14.4	14.233

where Y is the response (MB degradation); βo is the model intercept and βi is the linear coefficient, and Xi is the level of the independent variable. The significance of each examined variable depending on its nature (i.e., positive or negative effect on the response) was evaluated by the main effect that was deduced from the statistical analysis.

#### Central composite design (CCD) method

The effect of five process factors concluded from PBD, namely NaNO_3_, molasses, MB concentrations, bacterial inoculum size and pH, on the MB degradation was investigated and optimized using central composite design (CCD). The experimental matrix consisted of 32 trials, and each independent variable was examined at five different levels (− 2, − 1, 0, 1, 2). The trials were prepared in duplicate by adding 100 mL of media in 250 mL flasks and incubated under orbital shaking conditions (150 rpm) at 30 °C. The statistical calculation describing the relationship between the coded and actual values was represented by Eq. (2):

2 $${\text{Xi}} = {\text{Ui}} - {\text{Ui}}_{0} /\Delta {\text{Ui}}$$

where *Xi* is the coded value of the *i*th variable, *Ui* is the actual value of the *i*th variable, Ui_0_ is the actual value of the *i*th variable at the center point and ΔUi is the step change of variable. The second-order polynomial structured represented in Eq. (3):

3 $$Y \, = \, \beta_{0} + \beta_{{1}} X_{{1}} + \beta_{{2}} X_{{2}} + \beta_{{3}} X_{{3}} + \beta_{{{11}}} X_{{{11}}} + \beta_{{{22}}} X_{{{22}}} + \beta_{{{33}}} X_{{{33}}} + \beta_{{{12}}} X_{{1}} X_{{2}} + \beta_{{{13}}} X_{{1}} {\text{X}}_{{3}} + \beta_{{{23}}} X_{{2}} X_{{3}}$$

where *Y* is the predicted response; X_1_, X_2_, X_3_ are input variables which influence the response variable Y*; β*_0_, intercept; *β*_1_, β_2_ and *β*_3_ linear coefficients; *β*_11_, *β*_22_ and *β*_33_, squared or quadratic coefficients *β*_12_, β_13_, and *β*_23_ interaction coefficients**.**

#### Statistical analysis

The experimental matrices, the regression analysis for determining the analysis of variance (ANOVA), three-dimensional surface plots (3D), two-dimensional contour plots (2D) and optimizer tool were figured out using statistical software Minitab 14.0 (Minitab Inc., Pennsylvania, USA) [42, 43].

#### Verification of experimental model

The statistical model was validated by comparing MB degradation under conditions predicted by the statistical design and the basal media.

### MB decolorization assay

For each PBD and CCD-experiment, MB degradation was evaluated by measuring the absorbance of clear supernatants at the absorption maxima (λ max) of 665 nm in UV–Vis spectrophotometer (Labomed model). An un-inoculated control was run in parallel for each trial in both PBD and CCD matrix. The experiments were performed in triplicate and the average was considered. The discoloration efficiency was calculated based on the following equation [44]:

4 $$\mathrm{MB degradation }(\mathrm{\%})=\frac{\mathrm{Initial Absrobance}-\mathrm{Absorbance after degradation}}{\mathrm{Initial Absorbance}}\times 100.$$

### Enzymes activity assays

Under optimized conditions, the enzymes involved in MB-biodegradation, including manganese peroxidase (MnP), nitrate reductase (NR), tyrosinase, lignin peroxidase (LiP), NADH-DCIP reductase, azoreductase and laccase, were conducted to determine their role in this process as a function of time (12 h interval). The microbial biomass, which was utilized as a source of intracellular enzymes, was suspended in phosphate buffer (50 mM, pH 7.4) for sonication by Ultrasonic Disruptor (UD-200, Tommy, Tokyo, Japan) for 5 min at 40–60% amplitude and frequency of 20 kHz with 0.6 s pulse rate at 4 °C to complete disruption of the cells. The procured lysate was consequently centrifuged for 7 min at 4 °C at 10,000 rpm, the clear solution was used as crude enzyme for all assays as follows:

*Manganese Peroxidase (MnP) activity* was assayed according to method of [45] using phenol red at 30 °C. In brief, 100 µL of enzyme sample was incubated in a total volume of 1 m of reaction mixture containing 0.1 mg of phenol red, 25 mM lactate, 0.1 mM MnSO_4_, 1 mg of bovine serum albumin (BSA) in 20 mM sodium succinate buffer, pH 4.5. The reaction was initiated by the adding a final concentration of 0.1 mM H_2_O_2_ and stopped by adding 10% NaOH after 1 min of incubation, and absorbance was measured at 610 nm. Control samples from phenol red oxidation were included in the absence of MnSO_4_ from the reaction mixture. The activity of MnP activity was evaluated by subtracting the values of control samples from values of the enzyme which obtained in the presence of MnSO_4_.

*Nitrate reductase activity* was determined by measuring the liberating nitrite from reduction of nitrate with NADH as an electron donor. In brief, 0.3 mL of the enzyme sample was added to the reaction mixture containing 0.1 M NaNO_3_ and 25 mM NADH in 0.2 M K-Na phosphate buffer, pH 7.1. The reaction mixture was gently vortexed and incubated at 30 °C for 30 min. The reaction was completed, and the absorbance at 540 nm was measured [46].

*Tyrosinase activity* was determined according to method of [47] by using l-tyrosine and l -DOPA as substrates. In brief, about 0.1 mL of the enzyme solution was added to an aliquot to a reaction mixture containing 1 mM l-tyrosine and l-DOPA in 0.1 M sodium phosphate buffer (pH 6.8), and the formation of dopachrome is monitored by reading the reaction absorbance at 475 nm.

*Lignin peroxidase (LiP) activity* was assayed according to method described by [48]. In brief, 0.1 mL of the enzyme sample was added to a reaction mixture containing 100 mM n-propanol, 250 mM tartaric acid, and 10 mM H_2_O_2_ in 10 mM sodium tartrate buffer, pH 3.5. The enzyme activity was monitored by measuring the released propanaldehyde at 300 nm. The molar extinction coefficient of n-propanol was 0.02/mM/cm.

*The NADH-DCIP reductase activity* was assayed by monitoring NADH and DCIP reduction at 590 nm for 60 s. In brief, 0.1 mL of enzyme sample was added to the reaction mixture containing 50 μM DCIP and/or 50 μM NADH in 50 mM phosphate buffer, pH 7.4. The enzyme activity was determined using the extinction coefficient of 90/mM/cm [49].

*Azoreductase activity* was assayed according to [50]. In brief, 0.1 mL of enzyme sample was added to the reaction mixture containing 100 mM NADH, 50 mM methyl red (MR) in 50 mM potassium phosphate buffer, pH 7.5. The decrease of MR concentration in the sample was monitored by measuring the absorbance at 430 nm for 270 s. The enzyme activity was determined by using an extinction coefficient of 23.36/mM/cm.

*The laccase activity* was calculated according to [51]. In brief, 0.1 mL of enzyme sample was incubated at room temperature with 10 mM Guaiacol in 100 mM sodium acetate buffer, pH 5.0. The change in the reaction absorbance was measured at 470 nm for 10 min of incubation.

Generally, to determine specific activity of the enzymes (units/min/mg protein), the protein content in all examined specimens was estimated by reading the OD of samples at 280 nm or by protocol of [52] using BSA as a standard protein**.**

### Analysis of metabolites generated from MB detoxification

The metabolic by-products formed after MB degradation, on optimized medium incubated with the developed consortium under study, were analyzed and compared with MB-control before degradation. Upon completion of degradation process, 100 mL of degraded and MB-control mixtures were centrifuged at 10,000*g* for 10 min. The obtained supernatant was extracted by addition of equal volume of ethyl acetate, dried over anhydrous sodium sulfate and evaporated in a rotary evaporator. The final crystals were dissolved in a small volume of distilled water, filtered through a 0.22 µm membrane and subjected to the following analysis [13].

#### UV−visible spectrophotometric analysis

The change in UV−visible spectra of extracted solution before and after MB degradation was monitored by visible spectrophotometer (Labomed model). About 2 mL of supernatant was drawn and scanned at absorption spectrum from 200 to 800 nm.

#### Fourier-Transform Infrared (FTIR)

The biomass-free degraded metabolites and MB-control solutions were analyzed using Shimadzu FTIR-8400 S, Japan. The dehydrated and lyophilized samples were mixed with potassium bromide and pressed into discs, which were scanned over the wavelength (4000–400 cm^−1^) with a resolution of 4 cm^−1^ [53].

### Acute toxicity evaluation

#### Phytotoxicity bioassays with* Chlorella vulgaris*

The toxic effects of MB (200 mg/L) and its biodegradation by-product were assessed in comparison to the control group (without any treatments). Approximately 100 mL of Bold’s basal media (BBM) medium were inoculated with *C. vulgaris* cells in the presence of MB (200 mg/L), degradation by-products in both optimized media and basal media compared to the control group. The flasks were incubated under illumination for 4 days at 25 °C [54]. At the end of the incubation period, the algal biomass was collected by centrifugation at 15,000*g* for 15 min for pigment fractionation according to method mentioned by [55]. A fixed amount of biomass was heated at 70 °C in presence of methanol for 15 min. Thereafter, the processed debris was eliminated by centrifugation and the clear supernatant containing the pigments was diluted to a definite volume. The extinction coefficient was monitored using spectrophotometer against a blank of methanol at the wavelengths of 644, 663 and 452.5 nm, which corresponds to chlorophyll-a, chlorophyll-b and carotenoids, respectively. Considering the dilution factor, the content of pigment fractions was expressed in µg/mL algal suspension and calculated form the following equations [55]:

5 $$\mathrm{Chlorophyll \,a }= 10.3\mathrm{ E}663 - 0.918\mathrm{ E}644$$

6 $$\mathrm{Chlorophyll \,b }= 19.7\mathrm{ E}644- 3.87\mathrm{ E}663$$

7 $$\mathrm{Carotenoids }= 4.2\mathrm{ E}452.5 - \left[0.0264\mathrm{ Chl}.\mathrm{ a }+ 0.4260\mathrm{ Chl}.\mathrm{ b}\right]$$

#### Cytotoxicity using human cell lines

Normal cell lines such as the human fetal lung cell line (Wi-38) and the adult African green monkey kidney cell line (Vero) were recruited to examine the toxicity of MB dye before and after biodegradation according to the procedure explained by [56]. Both examined cell lines were maintained in DMEM medium (Lonza, USA) containing 10% fetal bovine serum and sub-cultured for 2 weeks before assay using trypsin EDTA (Lonza, USA). Their viability and counting were calculated by trypan blue stain and hemocytometer. Wi-38 and Vero were seeded in 96 well culture plate as 1 × 10^4^ cells per well and incubated at 37 °C in 5% CO_2_ incubator. Subsequently, the cells were exposed to MB (200 mg/mL) and their degraded by-products, after 24 h for cell attachment. However, upon 72 h incubation in 5% CO_2_ incubator, 20 µL of MTT solution (5 mg/mL) was supplemented to each well and incubated at 37 °C for 4 h in 5% CO_2_ incubator. MTT (Sigma, USA) solution was removed and the insoluble blue formazan crystals trapped in cells were solubilized with 150 µL of 100% DMSO at 37 °C for 10 min. The absorbance of each well was screened with a microplate reader (BMG LabTech, Germany) at 570 nm to determine the percentage of cell viability**.**

### Application of consortium in the treatment of real effluents

#### Consortium immobilization and characterization

The detoxification potential of an immobilized consortium was examined in MB-removal from effluents collected from different sources and artificially contaminated with MB. In a comparative manner, equivalent microbial biomass was suspended in 20 mL of optimized slurry (COS) and distilled water (CDW) and entrapped in alginate beads. In 20 mL of 3% (w/v) sodium alginate solution, both mixtures were homogeneously blended and dropped into 150 mL of 2% (w/v) chilled sterile CaCl_2_ by gravity using syringe and needle. The drops of biomass-alginate mix were gelled to form 4 mm diameter beads. Both COS and CDW-gelled spheres were maintained in sterile CaCl_2_ (2%) for 2 h, under aseptic conditions, at room temperature to complete gel hardening. For subsequent use, the beads were washed three times by sterile distilled water to remove any residues [57]. Scanning electron microscope (SEM-JEOL JEM-1230, Japan) was utilized to visualize and characterize the internal morphology of beads before and after treatment.

#### Bioaugmentation study of COS and CDW alginate beads in MB-contaminated effluents

The biodegradative performance of COS and CDW either freely-suspended or immobilized beads in MB-bioremediation process from various effluents was tested. Industrial wastewater was collected in March-2021, from the discharge of line-industrial zone-Borg El-Arab, Alexandria Egypt; whereas, the municipal wastewater effluent was collected from discharge line of Sewage treatment unite-26 K, Alexandria Egypt at february-2021. Both effluents were studied as a modular bioaugmentation tool. According to standards, the quality criteria of both specimens, including chemical, physical and biological were estimated. In 600 mL MB-artificially contaminated effluents, equal consortium lawns in both states were inoculated in both samples. In addition, plain (non-immobilized) alginate spheres were inoculated in separate flasks to detect the absorption percentage. The flasks were incubated as previously mentioned. The variation in UV–visible spectra of MB solution at concentration of 200 mg/L was checked spectrophotometrically (λ max ~ 665 nm) at 12 h time interval for 10 days The removal percentage of MB was calculated as represented in Eq. 4 [58].

## Results

### MB-degraders selection and compatibility assessment

In this study, among 35 bacteria, 6 actinomycetes, 7 fungi and 3 yeast strains, one yeast and two bacterial strains were selected based on the highest value of discoloring area around the colonies. The selected stains are *Rhodotorula sp., Raoultella planticola;* and *Staphylococcus xylosus.* Their 16S and 18S-rDNA genes were formerly identified and submitted in *GenBank* under accession numbers MK551748, MZ312359, MT940226, respectively. Despite they were screened from different environmental habitats with different contamination content. Some of them exhibited characteristic bioremediation capabilities [16]. The diversity of their phylogeny, namely *Basidiomycota, Proteobacteria*, and *Firmicutes*, revealing their metabolic performance to utilize and detoxify xenobiotic compounds. However, their amalgamation in single system based substantially on absence of antagonism, which appeared vividly through compatibility test. As pointed out by [59], the cooperative interaction among different strains in the consortium expedites and enhances their performance.

Obviously, high removal rate was recorded by our examined consortium upon using nutritive complex media, such as nutrient broth, supplemented by 200 mg/L of MB, which reached to 95% removal at 20 h. Nonetheless, from economical point of view, it is not applicable. However, the bioremediation process for xenobiotic pollutants is serious and complex and is regulated by nutrient supplementation, microbial species requirements, nature and concentration of pollutant [60]. Thus, it is plausible to optimize MB-bioremediation process in efficient, applicable and economic manner. Herein, MSM media was utilized to simulate the real effluents that are devoid from easily available nutrients. To enhance the performance of bioremediation process, the agro-industrial waste, viz molasses, was utilized as carbon source. Remarkably, molasses is a waste liquor produced form sugar manufacturing; it is highly nutritive raw material commercially employed in several industries [48, 49] Subsequently, the optimization process of MB-detoxification was designed by using statistical methods, including Plackett–Burman design (PBD) and response surface methodology (RSM).

### Optimization of MB detoxification conditions

#### Screening of significant parameters affecting the MB removal via PBD

In this study, PBD was utilized to determine the most significant independent variables to optimize MB degradation process. As demonstrated in Table 1, there was a notoriously variation that ranged from 1.7% (trial number 5) to 37.7% (trial number 2), implying the vital role of optimization process in enhancing MB degradation process. The multiple linear regression coefficient of the model was analyzed by MINITAB 14 via the student's t-test. Table 2 elucidated the coefficient of each variable (i.e., the effect of this variable on MB degradation and also their p-values). In general, p-value indicates the significance of each independent variable in the design. Where, the larger the magnitude of the t-value and the smaller p-value (prob > F < 0.05), the greater the significance and the influence of the corresponding coefficient term on the response [61]. Based on the calculated p-value, MB concentration (p-value, 0.017; confidence level = 98.3%), pH (p-value, 0.022; confidence level = 97.8%), molasses (p-value, 0.027; confidence level = 97.3%), inoculum size (p-value, 0.032; confidence level = 98.3%) and NaNO3 (p-value, 0.037; confidence level = 96.3%) were media ingredients that significantly influenced the MB degradation process.

**Table 2:** Statistical analysis of PBD showing coefficient, t-test values and *p*-values of variables influencing MB degradation

Term	Coef	SE Coef	T	p
Constant	9.5833	0.1667	57.5	0.011
NaCL	− 1.55	0.1667	− 9.3	0.068
MgSO_4_	− 2.0667	0.1667	− 12.4	0.051
KH_2_PO_4_	− 1.8333	0.1667	− 11	0.058
K_2_HPO_4_	0.6333	0.1667	3.8	0.164
NaNO_3_	2.9	0.1667	17.4	0.037
Molasses	3.9333	0.1667	23.6	0.027
MB conc	− 6.1	0.1667	− 36.6	0.017
Inoculum size (%)	3.2667	0.1667	19.6	0.032
Incubation time (days)	1.0833	0.1667	6.5	0.097
pH	− 4.9167	0.1667	− 29.5	0.022
	R^2^ = 99.9		R^2^ (adj) = 99.72	

From the standard analysis of variance (ANOVA) summary computed from experimental PBD tests, the model was highly significant evident of the low probability value [p-value = 0.04] (Table 3). In addition, the overall performance of the model was assessed by the coefficient of determination (R^2^) and the adjusted-R^2^ (adj-R^2^) value, which should be in reasonable agreement with R^2^ value (less than 2%) [51]. As indicated by [27], the stronger model with better prediction of response takes place by R^2^ value closer to 1. In the current study, the model R^2^ and adj-R^2^ values recorded for MB degradation were 99.9% and 99.7%, respectively. These results indicate that 99.9% of the variability of the data can be explained by the model, and there is only a 0.1% chance, which could be due to noise. Besides, the adj-R^2^ value was high proving the accuracy of the model and a high correlation between the predicted and the experimental values as observed in Table 1. All such items revealed a satisfactory adjustment for calculation of MB degradation. From ANOVA analysis, the first order model for MB degradation was fitted to the results obtained from the 12 experiments as the following equation (Eq. 8):

8 $$\begin{aligned} {\text{MB degradation }}\left( \% \right) & = \, 0.{9}.{58 }{-}{ 1}.{\text{55 NaCL }}{-}{ 2}.0{\text{6 MgSO}}_{{4}} {-}{ 1}.{\text{83 KH}}_{{2}} {\text{PO}}_{{4}} + \, 0.{\text{63 K}}_{{2}} {\text{HPO}}_{{4}} \\ & \quad + { 2}.{\text{9 NaNO}}_{{3}} + { 3}.{\text{93 Molasses }} \\ & \quad {-}{ 6}.{\text{1 MB }} + { 3}.{\text{2 inoculum size }} + {1}.0{\text{8 incubation time }} - { 4}.{\text{9 pH}} \\ \end{aligned}$$

**Table 3:** ANOVA for the quadratic model of MB degradation (%) by yeast-bacterial consortium

Source	DF	Seq SS	MS	F	P
Main Effects	10	1290.54	129.05	387.16	0.04
Residual Error	1	0.33	0.33		
Total	11	1290.88			

For the subsequent optimization step (central composite design CCD), all independent variables with a positive effect on MB degradation were maintained constant at their high level, and those factors negatively affect were kept at their low level.

#### Central Composite Design (CCD) for optimization of MB degradation

At this stage, the most of significantly influenced variables on MB degradation, identified by PBD, were further optimized by CCD. Where, the possible interactions among the significant parameters and their accurate evaluation of optimum levels were determined. Through 32-run matrix, MB, NaNO_3,_ molasses concentrations, pH and inoculum size were examined in five-level CCD, consisting of 16 factorial (cubic points), 10 axial or star points (points having an axial distance to the center of (α = ± 2), and 6 replicates of center points, as the risk of missing non-linear relationships in the middle of the intervals has to be minimized and the repetition allows determining confidence intervals [41]. The 32 trials, concentrations of tested factors at their coded and actual levels along with the experimental, predicted responses and studentized residuals are represented in Table 4. As noted, MB degradation differed considerably among the experimental runs displaying maximum response at trial 23 (factorial point) with 60.8%MB degradation and minimum response with 0.77% at trial 14 (axial point), reflecting the interactive effect of different factors with different levels**.**

**Table 4:** Central composite design (CCD) representing matrix and MB degradation by yeast-bacterial consortium as a function of NaNO_3_, molasses, MB concentrations, bacterial inoculum size and pH along with the predicted response, residuals and concentrations of variables at each level

Run Order	NaNO_3_ gm/L	Molasses μL/L	MB gm/L	Inoculum size (%)		pH	Experimental MB degradation (%)	Predicted MB degradation (%)	St. Residual
1	0	0	0	− 2		0	8.16	8.423	− 0.36
2	0	0	0	0		0	6.01	5.27	0.71
3	0	0	0	0		2	29.16	29.961	− 1.11
4	1	1	1	1		1	19.2	19.123	0.19
5	0	0	0	0		0	4.84	5.27	− 0.41
6	0	0	− 2	0		0	36.86	35.754	1.53
7	− 1	− 1	1	− 1		− 1	1.08	1.228	− 0.37
8	0	2	0	0		0	17.21	17.298	− 0.12
9	− 1	1	1	1		− 1	1.66	1.895	− 0.59
10	0	0	0	0		0	4.89	5.27	− 0.36
11	1	− 1	− 1	− 1		− 1	6	6.789	− 1.99
12	1	1	− 1	1		− 1	22.22	23.096	− 2.21 R
13	− 1	1	− 1	− 1		− 1	3.92	4.161	− 0.61
14	0	0	0	0		− 2	0.77	− 0.862	2.26 R
15	0	0	0	0		0	5.32	5.27	0.05
16	0	− 2	0	0		0	6.95	6.031	1.27
17	1	1	− 1	− 1		1	31.37	31.299	0.18
18	1	1	1	− 1		− 1	1.32	1.512	− 0.48
19	0	0	0	2		0	19.55	18.456	1.52
20	1	− 1	1	− 1		1	25.16	24.995	0.42
21	− 1	− 1	− 1	1		− 1	23.9	24.732	− 2.10 R
22	− 1	− 1	− 1	− 1		1	29.41	29.295	0.29
23	− 1	1	− 1	1		1	60.82	60.792	0.07
24	− 1	1	1	− 1		1	14.21	13.498	1.8
25	0	0	2	0		0	6.66	6.934	− 0.38
26	1	− 1	1	1		− 1	7	7.783	− 1.98
27	0	0	0	0		0	3.98	5.27	− 1.24
28	0	0	0	0		0	5.75	5.27	0.46
29	1	− 1	− 1	1		1	9.8	10.32	− 1.31
30	− 1	− 1	1	1		1	5.29	5.169	0.31
31	2	0	0	0		0	9.72	8.713	1.4
32	− 2	0	0	0		0	12.5	12.676	− 0.24

Variable		Coded levels/experimental values			
	− 2	− 1	0	1	2
NaNO_3_ (gm/L)	0.3	0.4	0.63	2	5
Molasses (μL/L)	100	200	300	500	1000
MB (gm/L)	0.2	0.25	0.3	0.35	0.4
Inoculum size (%, CFU/mL)	1% (10^8^)	3% (10^10^)	5% (10^12^)	10% (10^14^)	20% (2*10^14^)
pH	4	4.5	5	6.5	7.5

##### Multiple regression analysis and ANOVA

**Fig. 1:**
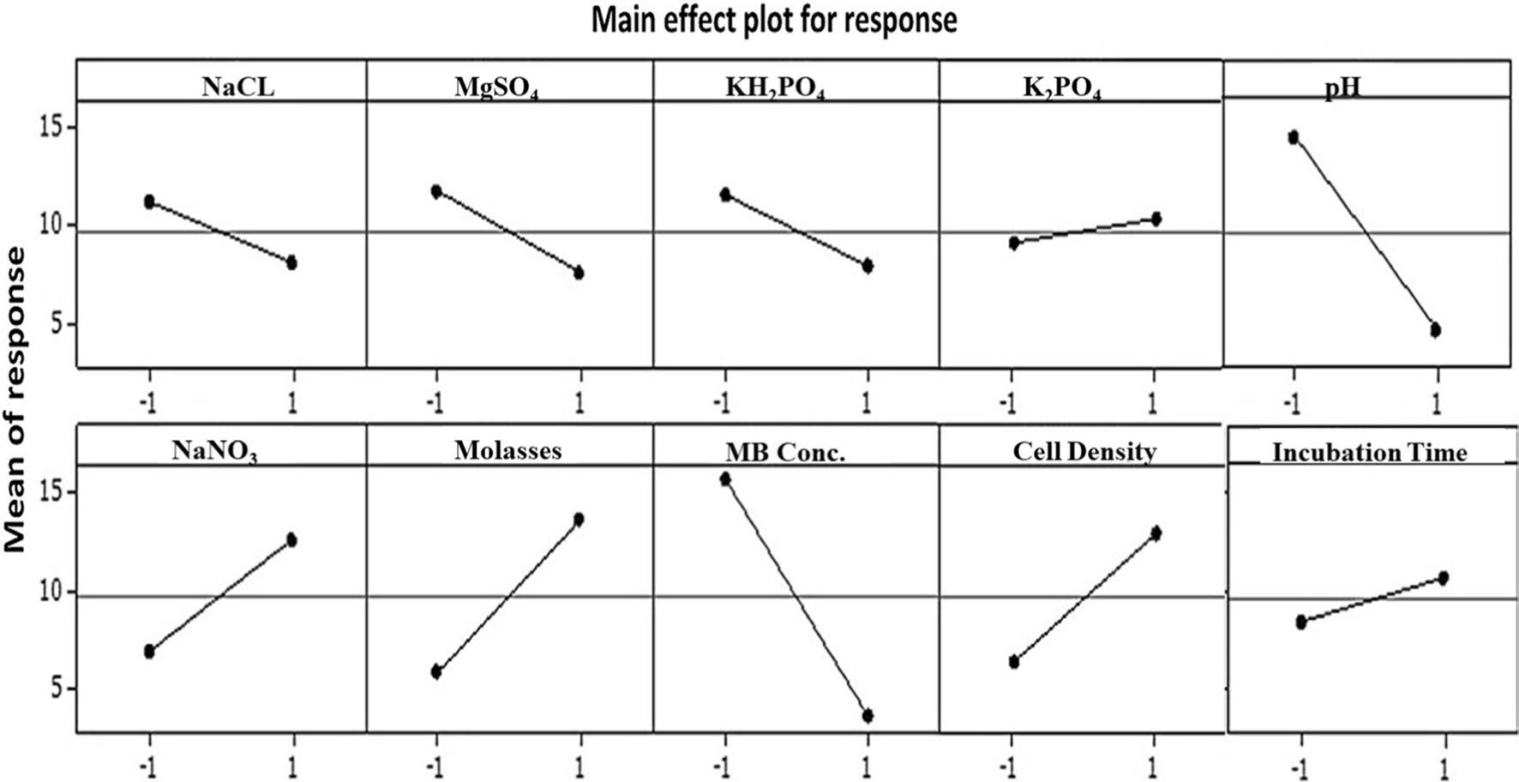
elucidates the main effect plot of each examined variable on MB degradation. The main effects characterize the deviations of the average between high and low levels for each variable. In case of the main effect of a factor is positive, the response (MB degradation) increases as the factor is changed from low to high level and is clearly observed in NaNO_3_, molasses and inoculum size. Whereas pH and MB lines deviated substantially from high to low level confirming its effect in low value on increasing MB degradation. Other factors such as K_2_HPO_4_, NaCl, KCL, MgSO_4_, 7H_2_O and incubation time exhibited neglectable effect on response, with the lines deviating by a very small degree from the horizontal baseline.

The results of CCD of MB degradation by the examined consortium were analyzed by multiple regression statistical analysis and ANOVA (analysis of variance) as summarized in Table 5. The parameters of statistical regression analysis, determination coefficient (R^2^) value, adj-R^2^ value, lack-of fit, model F-value and p-value, were calculated to determine the model reliability. Initially, the goodness of fit of the model was evaluated by R^2^ and adj-R^2^. Regarding R^2^, it is the ratio of response variance attributable to the model rather than to the random error; the regression model with a high R^2^ value higher than 80% indicates a good fit of a model. Besides, the adjusted-R^2^ should approximate that of R^2^ (≤ 2%) as refereed by [43]. The MB degradation regression model has R^2^ value = 99.7 (Table 2), indicating that 99. 7% of variations in the degradation percentages of MB dye were assigned to the independent variables and the model couldn’t describe just 0.3% of the total variations. Additionally, the regression model recorded adj-R^2^ = 99.3%, which seems to be in good agreement with R^2^, reflecting perfect coordination between the predicted and observed values of MB degradation; hence, the model of this study is optimal within the range of experimental parameters to predict an efficient MB degradation. However, the lack of fit test which designates the variation in the data around the fitted model, calculated by 0.089 as inferred by ANOVA (Table 3). Remarkably, insignificant lack-of-fit reveals a good model [62] (Fig. 1).

**Table 5:** Estimated effect, regression coefficients and corresponding *T* and *P* values in addition to ANOVA analysis for the optimization of MB degradation using CCD

Term	Coef	SE Coef	T	*p*		
Constant	5.2702	0.454	11.608	0		
NaNO_3_ (gm/L)	− 0.9908	0.2324	− 4.264	0.001		
Molasses (μL/L)	2.8167	0.2324	12.122	0		
MB (gm)	− 7.205	0.2324	− 31.008	0		
Inoculum size (%)	2.5083	0.2324	10.795	0		
pH	7.7058	0.2324	33.164	0		
(NaNO_3_)^2^	1.356	0.2102	6.452	0		
(Molasses)^2^	1.5985	0.2102	7.606	0		
(MB)^2^	4.0185	0.2102	19.12	0		
Inoculum size (%)	2.0423	0.2102	9.717	0		
(pH)^2^	2.3198	0.2102	11.037	0		
NaNO_3_* Molasses	0.3263	0.2846	1.146	0.276		
NaNO_3_* MB	4.9438	0.2846	17.372	0		
NaNO_3_* Inoculum size	− 3.0425	0.2846	− 10.691	0		
NaNO_3_* pH	− 1.8862	0.2846	− 6.628	0		
Molasses * MB	− 3.21	0.2846	− 11.28	0		
Molasses * Inoculum size	4.2963	0.2846	15.097	0		
Molasses * pH	4.05	0.2846	14.232	0		
MB * Inoculum size	− 3.4163	0.2846	− 12.005	0		
MB * pH	− 1.41	0.2846	− 4.955	0		
Inoculum size * pH	− 2.9688	0.2846	− 10.432	0		

Source	Df	Seq SS	Adj SS	Adj MS	*F*	*p*
Regression	20	5422.02	5422.02	271.101	209.22	0
Linear	5	3035.98	3035.98	607.195	468.61	0
Square	5	706.06	706.06	141.213	108.98	0
Interaction	10	1679.98	1679.98	167.998	129.65	0
Residual Error	11	14.25	14.25	1.296		
Lack of fit	6	11.59	11.59	1.932	3.63	0.089
Pure error	5	2.66	2.66	0.532		
Total	31	5436.27				

**Fig. 2:**
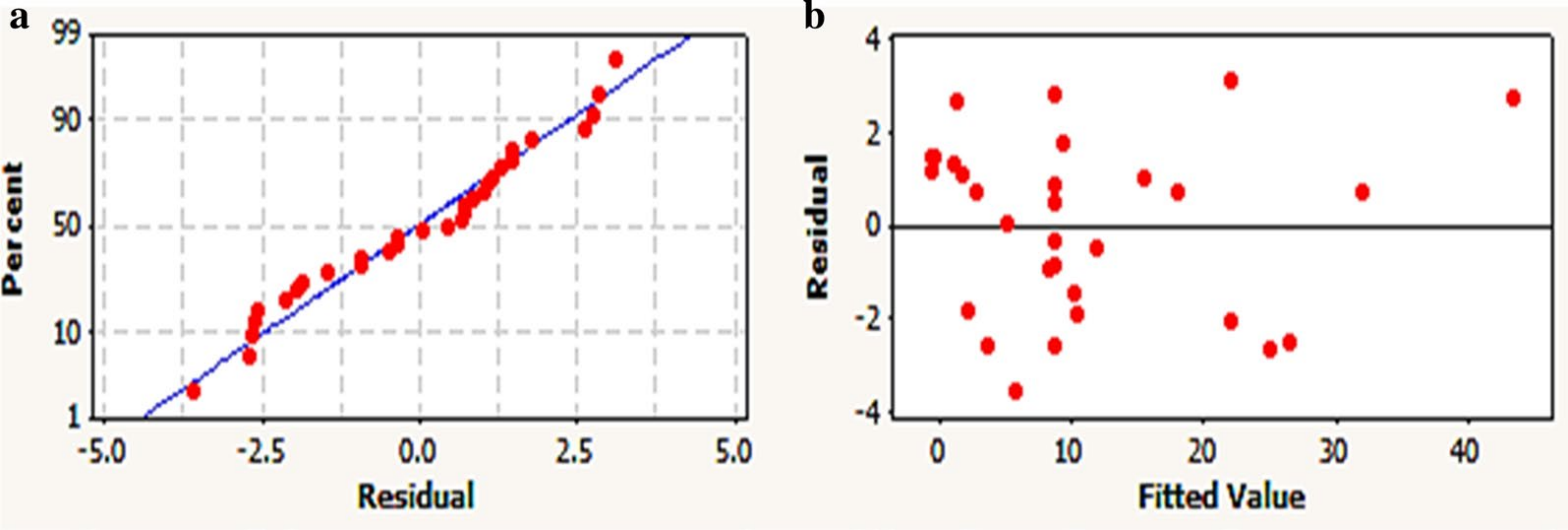
The normal probability plot of the residuals against MB degradation by the examined consortium determined by the second− order polynomial equation (**a**); Residual distribution against fitted values plot of MB degradation (**b**)

The significance of the model and also each parameter was assessed using P-values. As referred by [63], a low probability ‘p’ value (prob > F < 0.05) indicates a high significance of the analogue coefficient. As tabulated in Table 5, the ANOVA of the regression model of response (MB degradation) unveiled the higher significance of the model as evident from a small probability value (p < 0) with the calculated Fisher’s F test (F-value = 209.22). Furthermore, the normal probability plot confirmed the adequacy of the model (Fig. 2a). Where, the data points located closely along the straight line, implying that the residuals follow the normal distribution. Figure 2b shows the residuals versus the predicted values, indicating the random scattering of residuals around the center line without particular pattern. Moreover, the studentized residuals, which represent quotient of the residuals divided by the estimate of their standard deviation, fall within the adequate limit (less than ± 2) [41]. Interestingly, the entire parameters verified the model aptness, significance, adequacy and well-fitting to the experimental data.

Notably, the multiple regression analysis highlighted the impact of each variable linear coefficient, squared and their second-order interactions on MB degradation according to their sign (positive or negative) and their statistical significance (p < 0.05). It is clear that all linear, quadratic and interaction effect were significant except the interaction effect between NaNO_3_ and molasses by p-value = 0.276 (Table 5). It is evident that the interaction effect between the following pairs, NaNO_3_ and MB, molasses and inoculum size and molasses and pH can be described as synergistic effect. Their positive coefficients indicate a higher percentage of MB degradation upon elevating the concentration of both factors. Conversely, the correlation between the following combinations, NaNO_3_ and inoculum size, NaNO_3_ and pH and MB and pH considered as antagonistic. Where, a higher percentage of MB degradation was conjugated by increasing the level of one factor and decreasing the level of another one, demonstrated by the negative signs of their coefficients. However, the MB degradation as a response can be expressed in terms of second order polynomial equation (6).

6 $$\begin{aligned} {\text{MB degradation }}\left( \% \right) & = { 5}.{27 }{-} \, 0.{\text{99 NaNO}}_{{3}} + { 2}.{\text{81 molasses }}{-}{ 7}.{\text{2 MB}} \\ & \quad + { 2}.{\text{5inoculum size}} + { 7}.{\text{7 pH }} + { 1}.{35 }\left( {{\text{NaNO}}_{{3}} } \right)^{{2}} + { 1}.{59}\left( {{\text{molasses}}} \right)^{{2}} \\ & \quad + { 4}.0{1 }\left( {{\text{MB}}} \right)^{{2}} + {2}.0{4 }({\text{inoculum size}})^{{2}} + { 2}.{31 }\left( {{\text{pH}}} \right)^{{2}} \\ & \quad + \, 0.{\text{326 NaNO}}_{{3}} *{\text{ molasses }} + { 4}.{\text{94 NaNO}}_{{3}} *{\text{ MB }} \\ & \quad {-}{ 3}.0{\text{4 NaNO}}_{{3}} *{\text{inoculum size}}{-}{ 3}.0{\text{4 NaNO}}_{{3}} *{\text{inoculum size}} \\ & \quad {-}{ 1}.{\text{88 NaNO}}_{{3}} *{\text{ pH }}{-}{ 3}.{\text{21 molasses }}*{\text{ MB }} + { 4}.{\text{29 molasses }}*{\text{inoculum size}} \\ & \quad + { 4}.0{\text{5 molasses }}*{\text{ pH }}{-}{ 3}.{\text{41 MB }}*{\text{inoculum size}} \\ & \quad {-}{ 1}.{\text{41 MB }}*{\text{ pH}} - { 2}.{\text{96inoculum size}}*{\text{ pH}} \\ \end{aligned}$$

##### Graphical interpretation of the response surface model

**Fig. 3:**
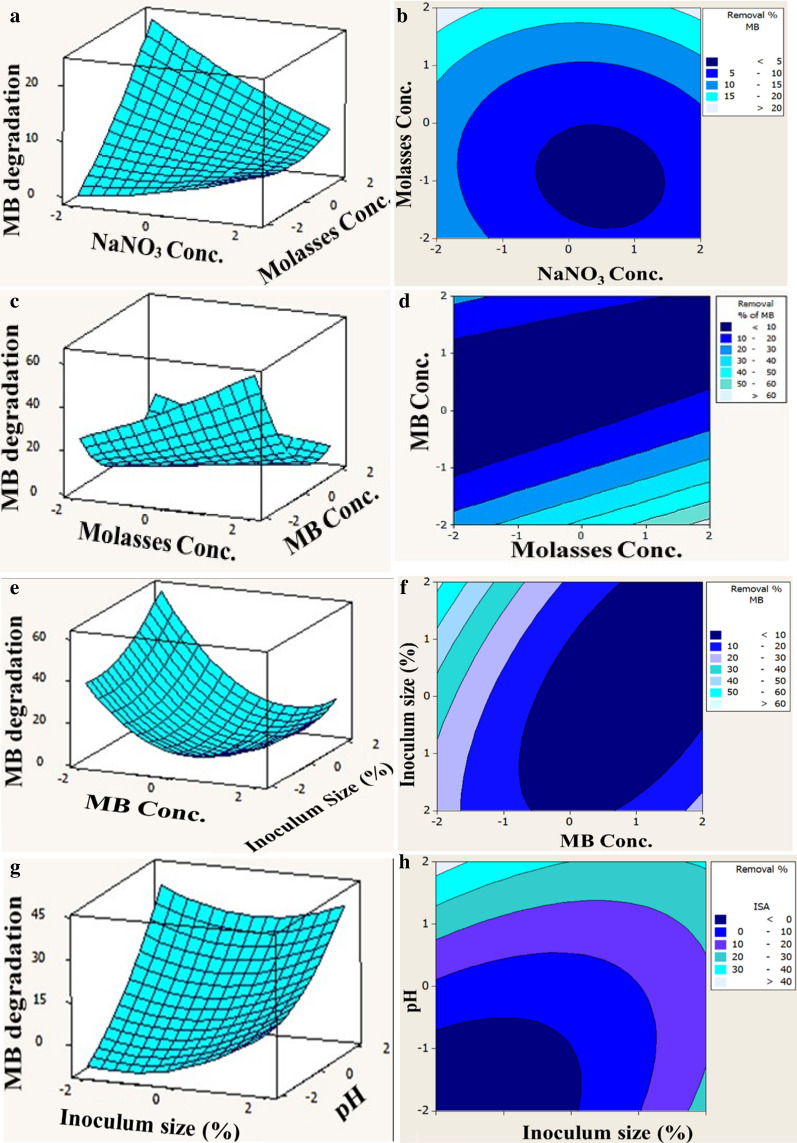
Three-dimensional-surface plot (left panels) and two-dimensional-Contour plot (right panels) illustrating the interactive effects of independent significant factors on MB degradation by consortium

The determination of the optimal concentrations of examined parameters and their interaction effect on MB degradation were plotted by using the three-dimensional surface plots (3D) and two-dimensional contour plots (Fig. 3). MB degradation was plotted on the Z-axis against two factors, while the rest factors were set at their zero level. Figure 3a, b demonstrate MB degradation as a response to NaNO_3_, molasses by maintaining the others at their midpoint levels. It showed that MB degradation increased gradually with the increase of molasses concentration and the maximum response obtained at the highest level of molasses (1000 μL) with varied NaNO_3_ concentrations from 0.3 to 5 g, indicating mutual interaction effect. While, more than 60% MB degradation was obtained at 1000 μL of molasses with the lowest concentration of MB (0.2 g) and a higher inoculum size represented by 20%, reflecting an antagonistic effect (Fig. 3c–f). In the same vein, the maximum degradation percentage of MB was achieved at the highest inoculum size with the lowest pH and vice versa Fig. 3g, h. Clearly, the 2D-contour plots of illustrated pairs denoted a significant interaction impact via their elliptical shape. As referred by [42], elliptical and saddle-shaped contour plots elucidate a significant interaction between examined factors; however, a circular contour plot unravels an insignificant interaction.

**Fig. 4:**
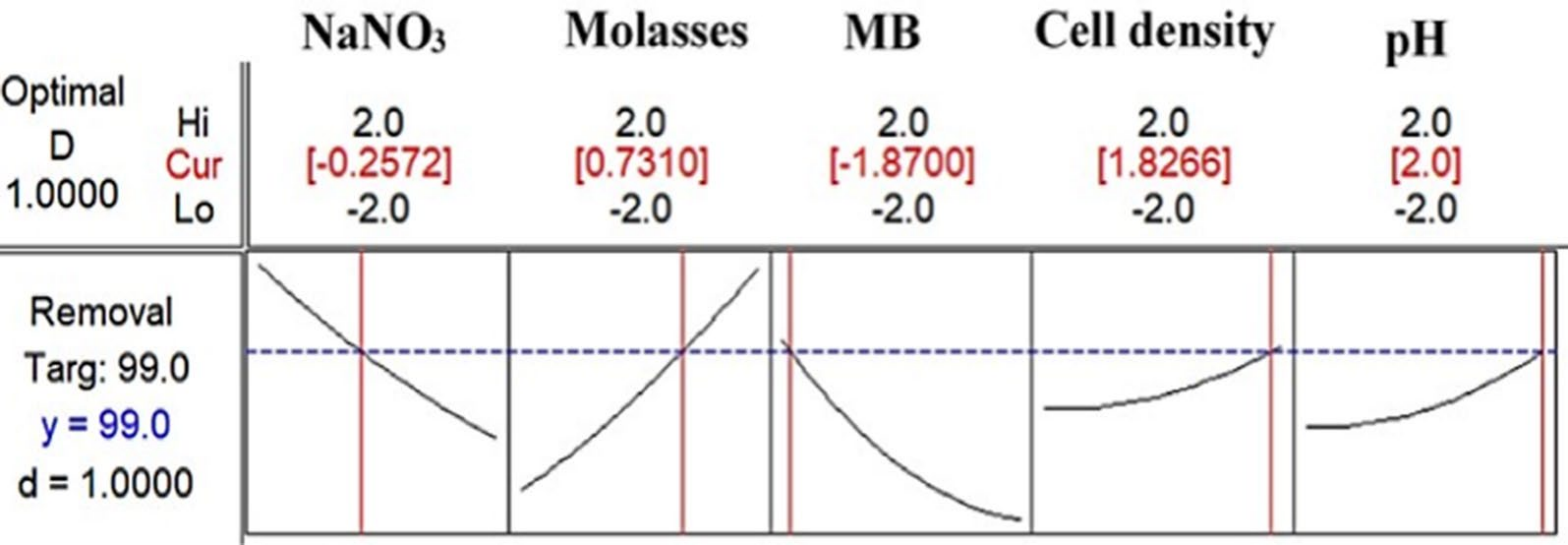
Response optimizer with desirability function for MB degradation with maximum goal and optimum levels of examined variables

In order to predict the maximum MB degradation, Eq. 6 was solved using the Optimizer tool in MINITAB 14.0 (Fig. 4), which estimates individual desirability by a desirability function. Such function identifies the adequate combination of independent parameters to procure the maximum response. Its range varied from zero (less accepted) to one (the accepted) [64]. Therefore, the highest percentage of MB degradation would be achieved at MB (0.2 gm/L), NaNO_3_ (0.525 gm/L)_,_ molasses (385 μL/L), pH (7.5) and inoculum size (18%).

##### Experimental verification of model

The validation of the results concerning MB degradation by yeast-bacterial corporation was assessed under optimum conditions predicted from the CCD and compared to the basal conditions. The optimization strategy led to threefold enhancement of MB from 29% (basal) to 91.5% (optimized).

### Enzymes activity assays

**Fig. 5:**
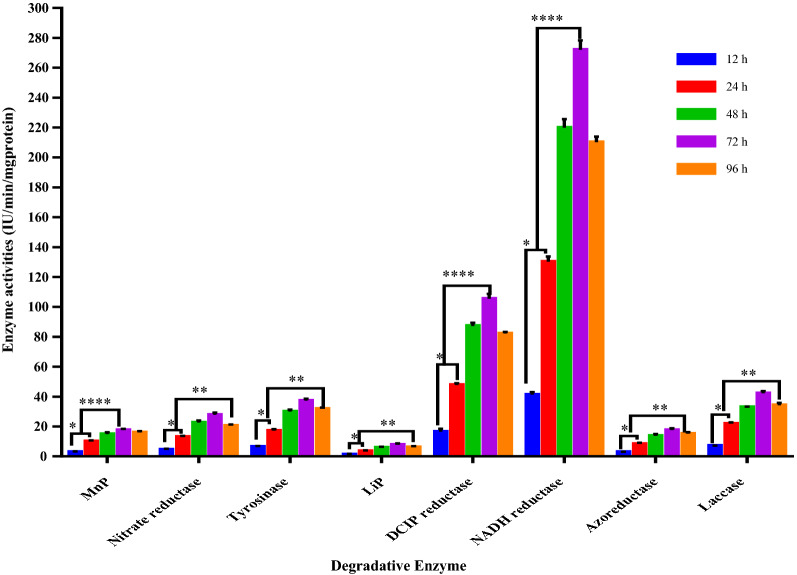
Quantitative bio-induction of eight different enzymes during degradation of MB dye by consortium of *Rhodotorula sp., R. planticola;* and *S. xylosus.* All values were expressed as mean ± SEM (standard error of mean), n = 3. The activity of degradative enzymes as a function of time with significance at p-value ≤ 0.025*, ≤ 0.01**, ≤ 0.0001****

Decolorization of synthetic dyes is essentially contributed to the interaction of the dye substrates with enzyme active sites leading to the generation of new degradable products of the synthetic dyes [65]. There are many degradative enzymes associated with dye decolorization such as laccases, reductases and peroxidases enzymes. In the present study, the degradative enzymes responsible for biodegradation of MB were estimated at 12 h interval for 96 h. The cell lysates of *Rhodotorula sp., R. planticola; and S. xylosus* consortium have displayed significant intracellular activities of 8 degradative enzymes including manganese Peroxidase (MnP), Nitrate reductase (NR), tyrosinase, NADH-reductase, DCIP-reductase, lignin peroxidase (LiP), azoreductase and laccase with its maximum activity after 72 h of incubation with MB as shown in Fig. 5. The activity of all tested enzymes was increased steadily with increasing the incubation time, which means that the induction of degradative enzymes was increased in time dependent manner during the MB-decolorization. The bacterial consortium showed promising production of intracellular MnP and LiP with maximum enzymatic activity estimated to be 17.59 ± 0.76 and 7.82 ± 0.85 IU/min/mg protein after incubation periods of 72 h, respectively. Maximum intracellular activities of nitrate reductase, DCIP-reductase, NADH-reductase and azoreductase were also assessed to be 28.36 ± 0.82, 104.52 ± 1.75, 274.04 ± 3.37 and 18.16 ± 0.47 IU/min/mg protein. Interestingly, both DCIP-reductase and NADH-reductase seemed to be induced with high activity during dye decolorization. Similarly, tyrosinase and laccase were found to be expressed during dye decolorization with maximum enzymatic activity of 36.78 ± 0.64 and 42.68 ± 0.80 at the exact time during decolorization process. Remarkably, all tested degradative enzymes were found to be repressed after complete MB decolorization.

### Analysis of metabolites generated from MB detoxification

#### UV−visible spectrophotometric analysis

**Fig. 6:**
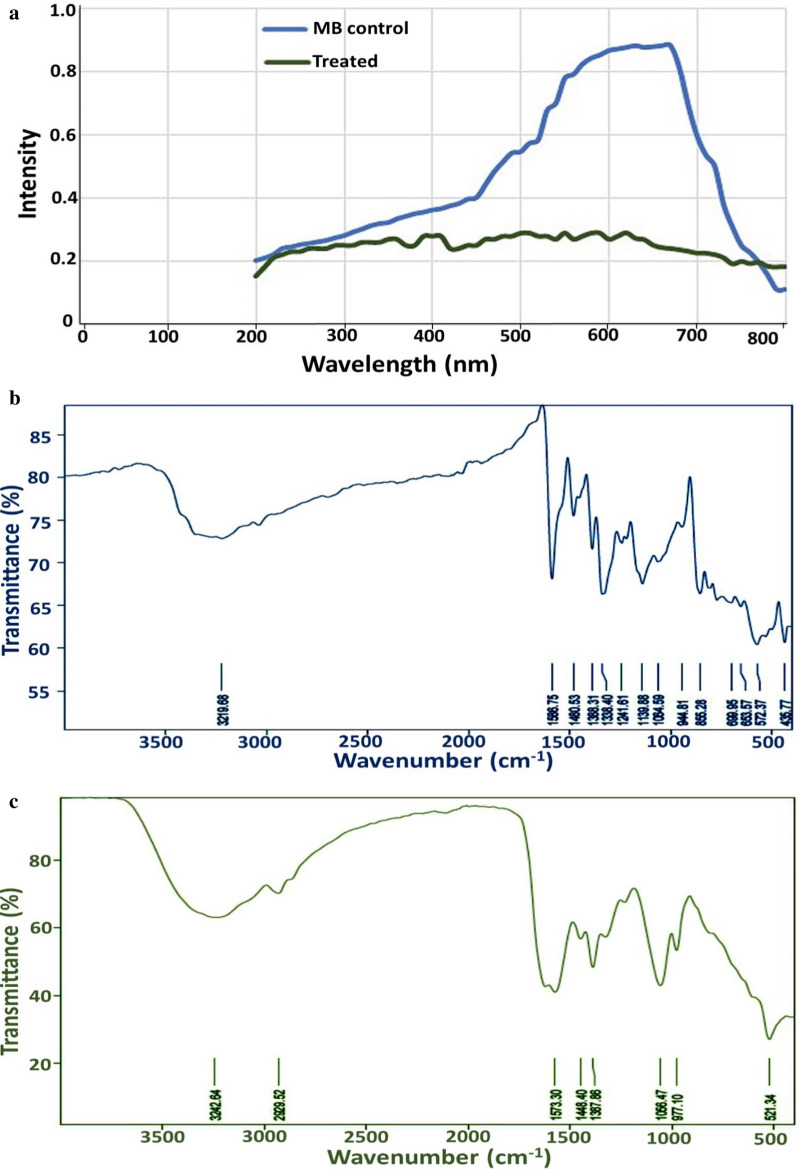
UV–Vis profile of MB before and after degradation by examined consortium (**a**); FTIR profile of MB before degradation (**b**); after degradation (**c**)

UV–vis spectral analysis of MB and its generated metabolites were monitored by a UV–vis spectrophotometer (Labomed model) in the spectrum range of (200–700 nm). As shown in Fig. 6a, a major peak appeared in the visible region at 665 nm in MB sample before treatment. However, upon degradation by the aid of yeast-bacterial consortium under optimized conditions, such peak vividly vanished.

#### FTIR

FTIR study attempted to identify the dye compounds and to confirm degradation by-products after the decolorization treatment process. The complete decolorization and demethylation of cationic MB was confirmed by comparing FTIR profiles of MB solution before and after treatment (Fig. 6b, c). In general, significant shifts in the number of characteristics peaks were observed in the spectrums of degradation supernatant. Concerning to the spectrum of the control including MB dye before treatment (Fig. 6b), several absorbance peaks were observed at 3220 cm^–1,^ 2920 cm^–1,^ 1570 cm^–1,^ 1480 cm^–1,^ 1140 cm^–1^ and 870 cm^–1^ which are belongs to MB dye molecules [56]. The peak at 3220 cm^–1^ was attributed to the overlapping of –OH and/or –NH stretching vibrations absorbance. The weak peak allocated at 2920 cm^–1^, confirmed the presence of –CH symmetric and asymmetric stretching vibrations of –CH_2_ groups. The band at 1570 cm^–1^ and 1480 cm^–1^ belonged to the stretching band of C‚O, C–N from the amide II and the symmetrical stretching band of carboxyl (–COOH), respectively. The strong peaks at 1140 cm^–1^ and 870 cm^–1^ indicated the bending band of N–H and C–N from the amide III band functional group. Additionally, the absorbance peaks at region of 830–560 cm^−1^ were attributed to the C–Cl stretching, which all characterize MB-FTIR profile [57]. Comparing this spectrum with that obtained after MB decolorization process (Fig. 6c), a significant reduction in FTIR peaks was observed in 1570 cm^–1^ to 870 cm^–1^ regions of metabolites suggested the absence of charged amines in the produced metabolites. Furthermore, peak shape for CHx stretching modes (3400–2900 cm^–1)^ after the treatment process is shaper than those at the MB spectrum before treatment, which indicates that the position of C-Hx was altered from –N(CH_3_)_2_ in MB to more flexible sites, for example, direct bonding to aromatic rings. A significant peak at 1660 cm^–1^ assigned to NH deformation, suggested the possible alkenes conjugation with CO. Moreover, peaks at 977 cm^–1^ and 1056 cm^–1^ for C–H deformation and absence of bands at of 830–560 cm^−1^ suggested the cleavage of MB dye molecule after decolorization process.

### Acute toxicity evaluation

#### Phytotoxicity bioassays with* C. vulgaris*

**Fig. 7:**
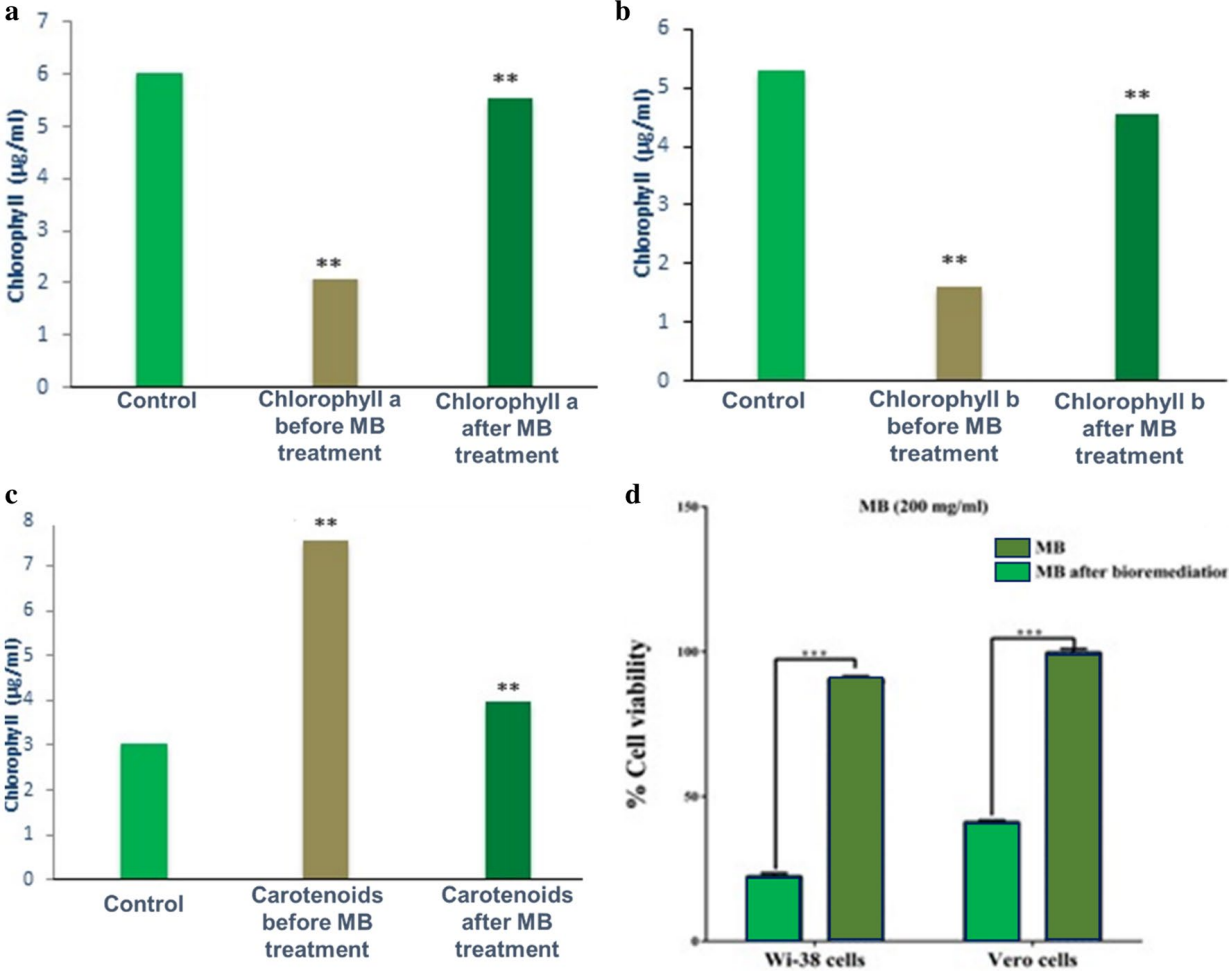
Acute toxicity evaluation. **a** Phytotoxicity of MB (200 mg/L) and bioremediated metabolites on *C. vulgaris* content of chlorophyll-a; **b** chlorophyll-b; **c** carotenoids and **d** Cytotoxicity in human cell lines. All values were expressed as mean ± SEM, n = 3 [**p ≤ 0.001, *** p ≤ 0.0001]

The influence of degradative by-products derived from optimized media containing 200 mg/L of MB, in comparison to MB without treatment and relative to the control (algal culture), was examined on photosynthetic performance of *C. vulgaris*. As observed in Fig. 7a, the reduction in chlorophyll-a in MB containing medium before treatment was 63.6%; however, it reached 8.2% in treated solution. Regarding chlorophyll-b, the results indicated that its content decreased by 69.6% in untreated solution and 14.1% in treated solution (Fig. 7b). On the contrary, the carotenoids content increased sharply prior to treatment with consortium by 149% and decreased sharply after treatment to reach 30% (Fig. 7c). Generally, the result revealed safety and non-toxic effect of MB-solution treated by *Rhodotorula sp., R. planticola* and *S. xylosus* consortium***.***

#### Cytotoxicity in human cell lines

The viability of Wi-38 and Vero cells was suppressed by 22.43% and 41.17%, respectively, after exposure to 200 mg/mL of MB dye as displayed in Fig. 7d. Whereases, it was improved to be 90.67% and 99.67%, respectively after exposure to the treated solution. Hence, the results revealed the highest and significant potency of microbial consortium to remediate MB and reduce its toxicity in both normal cell lines compared to dye alone (p < 0.0).

### Application of consortium in real effluents treatment

The detoxification efficiency of the examined consortium, in both freely-suspended state and immobilized state, towards bioremediation of MB from artificially contaminated municipal wastewater and industrial effluent, vis bioaugmentation, was investigated. Initially, the chemical, physical and microbial quality of both effluents were analyzed as listed in Table 6. As observed, the municipal wastewater recorded higher values in total nitrogen, phosphorous, sulfate, sulfide, B.O.D and total count, which could be attributed to the decomposition metabolites of carbohydrates, proteins, fats and vegetable fibers lignin which are derived from human diets; besides synthetic detergents, soaps and other synthetic organic chemicals. Whereas, industrial effluent recorded higher amounts of heavy metals, oil, C.O.D and phenolic compounds.

**Table 6:** Chemical, physical and microbial analysis of municipal wastewater and industrial effluents

Parameter	Industrial effluent	Municipal wastewater
	Concentration	
Total Nitrogen (mg/L)	131.24	723.2
T Phosphates P (mg/L)	0.34	83.1
T.D.S (mg/L)	510	238
Nitrate (mg/L)	25.87	374.9
Nitrite (mg/L)	3.1	62.2
Ammonia (mg/L)	0.194	253.3
Chloride	94.8	119.3
Carbonate (mg/L)	64.6	28.3
Sulfate (mg/L)	93.1	311.18
Sulfide (mg/L)	78.6	188.9
Phenol (mg/L)	104.6	0.04
Oil (mg/L)	165.9	28.3
B.O.D (mg/L)	111.6	321.9
C.O.D (mg/L)	310	344.6
Turbidity (NTU)	312.3	312.2
E.C (μs)	1123	601
Total count (CFU/L)	7.14 × 10^3^	8.74 × 10^9^
Calcium (mg/L)	132.04	47.69
Zn (mg/L)	23.4	0.14
Fe (mg/L)	31.6	1.8
Cr (mg/L)	9.6	< 0.01
Cd (mg/L)	3.5	< 0.01
Cu (mg/L)	15.2	< 0.01
Co (mg/L)	6.4	< 0.01
Ag (mg/L)	9.1	< 0.01
Pb (mg/L)	8.6	< 0.01

**Fig. 8:**
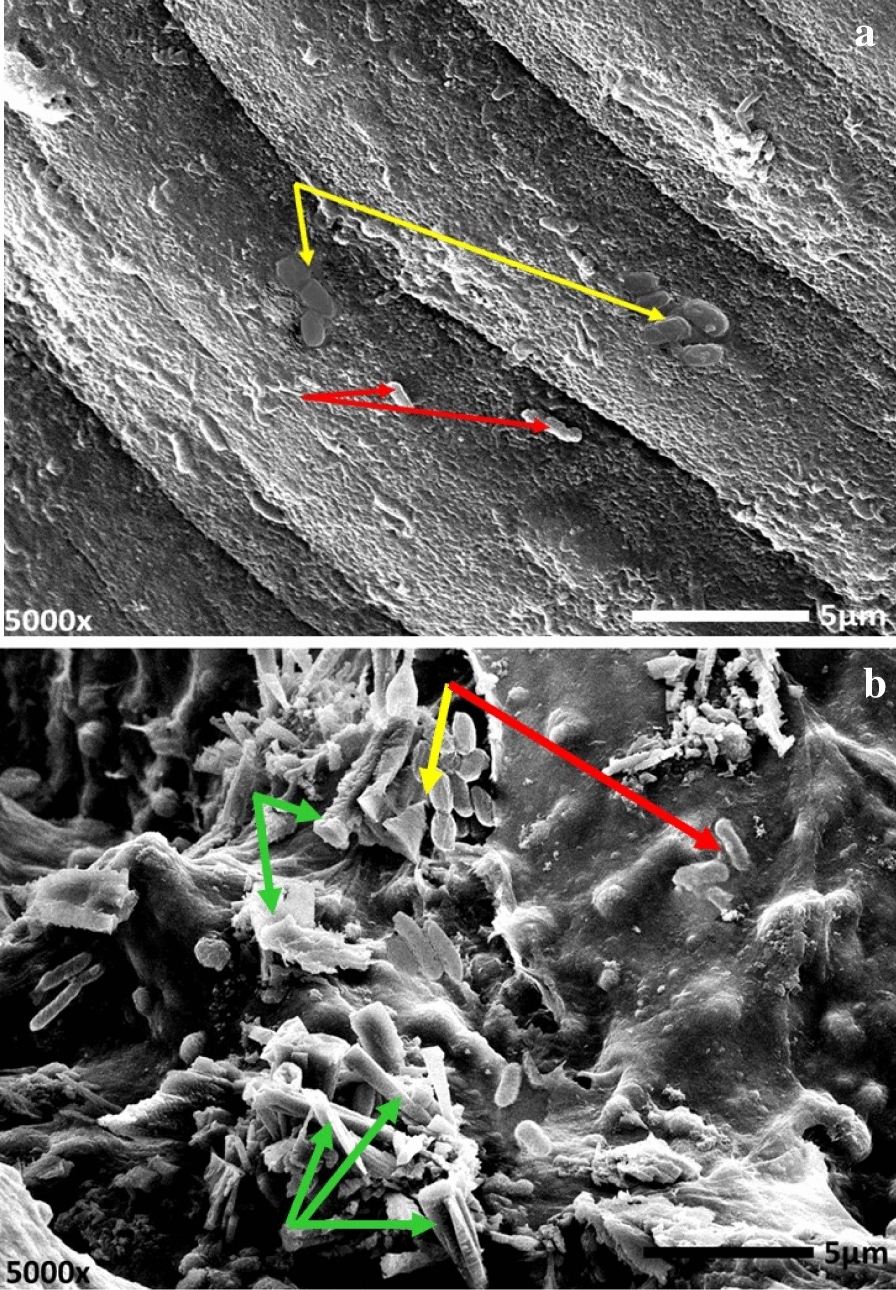
SEM micrograph demonstrating yeast-bacterial consortium immobilized in alginate matrix before treatment (**a**), after MB treatment (**b**). Yellow arrows: bacterial cells in consortium; Red arrows: yeast cells in consortium; Green arrows: MB molecules adsorbed on alginate spheres

**Fig. 9:**
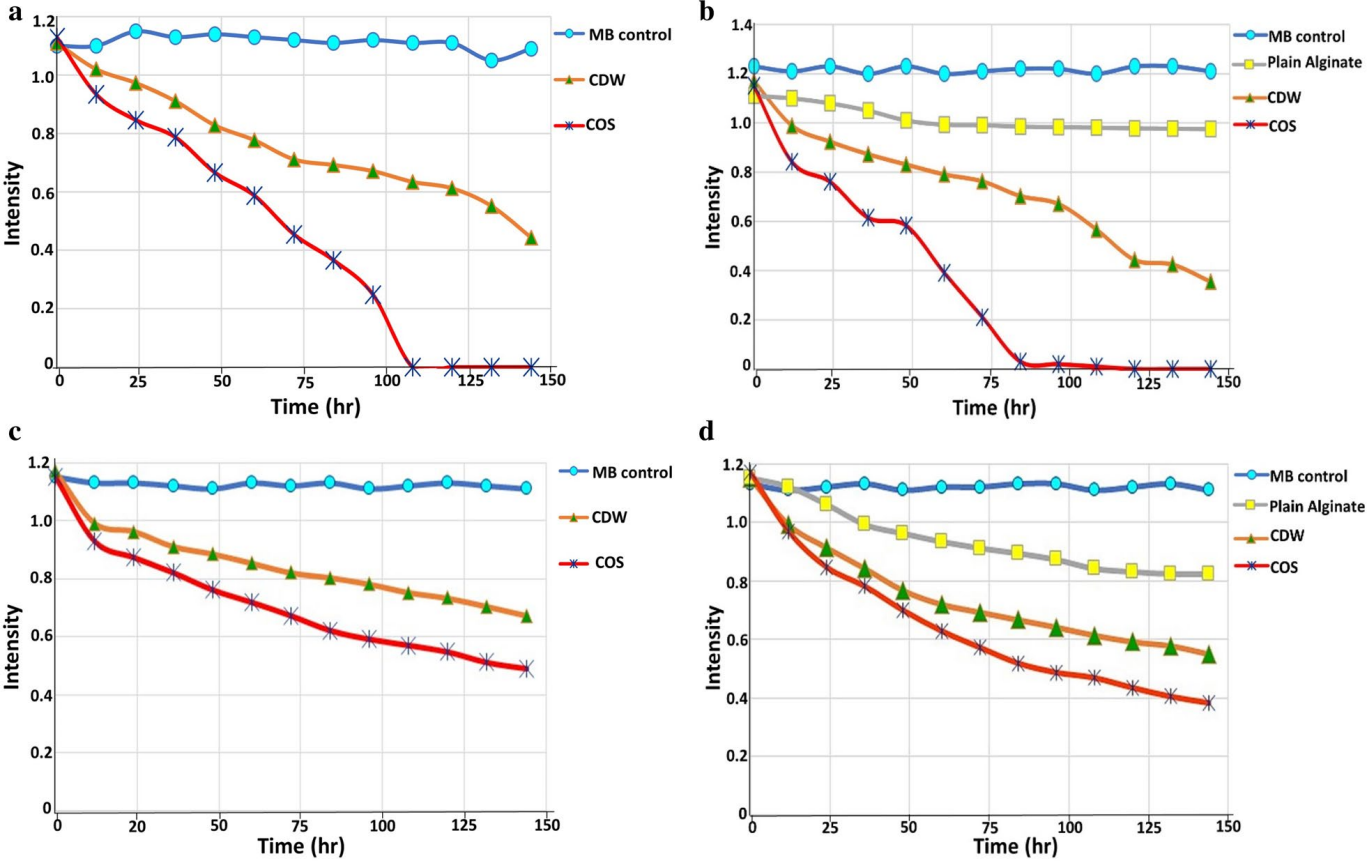
MB biodegradation from municipal wastewater and industrial discharges by examined consortium in optimized slurry and distilled water under freely suspended and immobilization states in bioaugmentation process **a** Freely suspended COS and CDW in municipal wastewater; **b** Immobilized COS and CDW in municipal wastewater; **c** Freely suspended COS and CDW in industrial effluent; **d** Immobilized COS and CDW in industrial effluent

Interestingly, by the dint of unique features provided by immobilization technique, the consortium in COS and CDW solutions was successfully immobilized in alginate spheres as illustrated in Fig. 8. As observed, both yeast and bacterial cells appeared clearly imbedded in alginate matrix (referred by arrows) before treatment (Fig. 8a). Besides, MB particles appeared adsorbed on the surface of immobilized beads as pointed out by green arrows in Fig. 8b. However, to investigate the efficacy of consortium to treat MB in bioaugmentation process, the equivalent inoculum in COS and CDW solutions, in both immobilized and freely suspended states, were employed to remediate 200 mg/L of MB from both effluents. Broadly, there were common features were observed as would be described. First, the plain alginate beads (without microbial cells), which run simultaneously as a control, exhibited absorptive capability ranged between 11.5 to 12.15%. Second, the bioaugmentation process followed time-dependent manner, indicated by gradual vanishing of MB as a function of the time as demonstrated in Fig. 9. Where, COS degraded 100 and 81 mg/L in of MB by immobilized states at 48 h for municipal wastewater and industrial effluent, respectively. However, upon 84 h and 144 h, a complete and 78.46% removal were implemented for municipal wastewater and industrial effluent, respectively. In the same context, the freely suspended state in COS eliminated 82.2 and 67.47 mg/L of MB at 48 h for municipal wastewater and industrial effluent, respectively; it was degraded entirely in municipal wastewater at 108 h. However, 114.7 mg/L of MB in industrial effluent was removed by 144 h. Third, more efficiency was achieved in a shorter period of MB bioremoval in COS in both states, namely freely suspended and immobilized, than that in the CDW form. This clearly indicated the complete degradation of MB in municipal wastewater at 84 h and 108 h for immobilized and freely-suspended states respectively. On the other hand, in CDW-augmentation system, 69.6 and 60.38% MB was removed by 144 h in municipal wastewater by immobilized and freely-suspended states, respectively. For industrial effluent, CDW-augmentation system removed 42.39 and 52.26% by 144 h compared to 57.39 and 78.46% elimination by COS for freely-suspended and immobilized states, respectively. Meanwhile, the immobilized consortium enhanced the diminishing percentage in the approximate range 9–22% increment than that exhibited by freely suspended state in both effluents. Additionally, in a separate trial, the indigenous microbiota removed about 9.2% of MB in municipal wastewater and 5.4% in industrial water during 144 h incubation (data not shown). Finally, the bioaugmentation performance of the examined consortium appeared to be superior in municipal wastewater discharge than the industrial one.

## Discussion

The discharge of hazardous effluents containing dyes from various industries into natural streams is one of major concerns regarding to health, environment and economics. However, the low degradative capability of indigenously occupying microbial residents often limits the bioremediation of azo dyes [17]. Hence, efficacious and economical approaches for wastewater treatment are essential to minimize or eliminate such toxicants to acceptable levels. In this study, the combination and mutual interaction of yeast, Gram-negative and Gram-positive strains enhanced the degradation performance of MB. Interestingly, several researches recorded the potency of *Staphylococcus* sp. and *Rhodotorula* sp. in azo dye decolorization [17, 58]. However, the decolorization ability of *R. planticola* was not studied before. Whereas, [66], reported the importance of *Klebsiella pneumoniae* in azo dye degradation, which is tightly-related genus to *R. planticola*. Noteworthy to mention the significant role of different yeast strains, such as *Candida tropicalis* and *Galactomyces geotrichum*, in treating several types of dyes than the bacteria. This could be attributed to their ability to cope xenobiotic compounds, due to their metabolic enzymes, and utilize their metabolic intermediates such as aromatic amines, which are toxic and suppressors to bacteria [19, 60]. Remarkably, the main mechanism to detoxify dyes via molds is biosorption, either by dead, live biomass or exopolysaccharide product [68]. So, yeasts are considered as advantageous over molds by their higher growth rates and increased growth density [69]. Yeasts are also adaptable microorganisms, with a greater ability to withstand harsh conditions such as high salt concentration, low pH, and high-strength organic wastewater (e.g. textile effluents) [70]. Nonetheless, the accurate designing and selection of consortium partners for better bioremediation under the exact experimental conditions is the challenge [39]. It is necessary to note that the right and successful combinatory state of microbial species could permit full degradation. In fact, individual bacterial or yeast species could attack MB from different positions and its intermediate by-product could be consumed by another species in such mixed-culture consortium; ultimately full decomposition of dye without accumulation of hazard residues [17]. However, more significantly these intermediate metabolites are not antagonistic to the other contributing species, which facilitates the bioremoval process efficiently. Notably, the presence of *Rhodotorula* sp. in the examined mixed community assisted virtually in expediting detoxification process by the virtue of synergistic interaction and compatible corporation in such mixed system. This characteristic was clearly noticed from the absence of antagonism in computability experiment implying the effectiveness of such consortium in MB bioremediation process. To our knowledge, no study has been reported addressing *Rhodotorula* sp. in mixed-coculture in MB degradation.

However, the higher removal rate was observed upon utilization of complex microbiological media, within 20 h, which is consistent with the findings of [67, 71], who used yeast extract (0.08–0.1 gm/L) and glucose (2–4 gm/L) in their trails to bioremediate azo dyes. From an economical point of view, it is necessary to develop substitute means to achieve more feasibility at industrial and environmental scales. Therefore, we utilized molasses as an inexpensive co-substrate for enriching and flourishing the examined mixed population in MB- bioremediation process. Mother liquor discarded as waste from sugar industry, full of micro- and macro- nutrients including, dissolved carbohydrates (mainly sucrose), crud proteins and vital minerals [72, 73]. Subsequently, the impact of molasses as a carbon source in addition to some nutritional and incubation factors, with different combinations, on MB degradation performance were studied simultaneously by the statistical design of experiment (DOE).

DOE as a collection of statistical and mathematical inference conducted to identify the perfect conditions for a multivariable system, construct and explore an approximate functional interaction between a response variable and a set of design independent variables techniques [74]. This approach was used to optimize environmental, incubation and nutritional conditions for improving and maximizing the performance of microbial activities without increasing the process cost [41]. It compensates the drawbacks of the main traditional approach, which is one-variable-at-time (OVAT). Via such single dimensional mean, the interaction effects among studied variables on the overall process were declined. Moreover, it is a time consuming, laborious practice and cost-effective. Recently, DOS has been utilized in media engineering to procure the optimal operating conditions for several purposes such as heavy metal removal [16], enzyme production [75] protein-drug production [74] and dye biosorption [63]. Initially PBD was utilized to screen the most significant factors, based on p-value, which were MB concentration, pH, molasses concentration, inoculum size and NaNO_3_ concentration. However, CCD was utilized as a second stage in optimization process to predict the optimal concentrations of significant factors that maximize removal of MB and also to give insight into their interactive influence on the bioremediation process.

Remarkably, the availability of the appropriate type and concentration of carbon/nitrogen sources boosted MB degradation by inducing the expression of oxidoreductive enzymes, which would favor MB decomposition to less complex metabolites as revealed by [76]. On the contrary, several studies stated the importance of using yeast extract, peptone and glucose for improving degradation efficiently [17, 24, 77, 78]. Such nutritive ingredients provide redox mediators such as NADH, which assist in energy generation, protein synthesis and subsequent metabolic activity. In agreement with our results, [79] reported an increase in Acid Orange azo dye degradation in media supplemented with NH_4_Cl. Besides, [80] showed that degradation of Red-2G by *Bacillus sp.* was improved upon the addition of external nitrogen source.

Herein, a successful selection of molasses and NaNO_3_, in small concentrations (385 μL/L and 0.525 gm/L), supported the biodegradation process of MB by their enriched nutritional values; fulfilling the required target in economic nutritional formulation. However, pH plays a crucial role in dye removal process as stated by [24]. It is closely related to functional groups that are present on the cell wall of microbial cells and also active sites of degradative enzymes. Where, inappropriate pH could distort the configuration of the enzyme active sites; subsequently it destroys the affinity of enzyme to dye, which ultimately adversely influences MB bioremediation. Moreover, net charge of both dye molecules and microbial cell are pH-dependent. Therefore, the changes in pH could result in undermining the interaction between cationic dye molecules and microbial biomolecules. In this context, [14] found that neutral pH (7) was the optimal value to procure the higher removal of MB by *Ralstonia eutropha*. Besides, *G. geotrichum* removed 71.5% of MB at pH 7 comparing to 6% and 12% obtained at pH 5 and 9, respectively [24].

Moreover, the proper cell-to-dye ratio governs the biodegradation performance of zo dye. Insufficient microbial biomass in the presence of a higher concentration of MB caused a lower rate of MB biodegradation. This can be explicated by the blocking and saturation of low number of microbial binding sites with highly available MB, thus, inhibition in bioremediation process [81]. In the same manner, [82] indicated the higher decolorization process of 1000 mg/L of MB by utilizing 185 mL/L inoculum size of *S. paucimobilis*, reflecting tolerance property of such strain. Also, [81] denoted the maximum removal of MB (5 mg/L) by *Stenotrophomonas maltophilia*, which diminishes in the removal process by increasing MB concentration to 20 mg/L. Obviously, the optimization process using PBD and CCD predicted the adequate concentration of each significant parameter to achieve maximum removal percentage of MB.

The pathway of MB biodegradation could be proposed on the basis of UV–Vis spectra and FTIR synchronizing with the data of enzyme assay. The disappearance of blue color from remediated solution came in parallel to the absence of absorption maxima at 665 nm, reflecting the transformation and destruction of MB structural moiety and its demethylation. Overall, the shifts in some peaks and the absence of others pointed out to that the produced metabolic by-products were devoid from amines, which were coincided with the activity of degradative enzymes, particularly DCIP-reductase and NADH-reductase. However, other ligninolytic enzymes such as LiP, MnP, and Laccase were also induced intracellularly, revealing the collective coordination of all such enzymes to decompose MB. The involvement of NADH-DCIP reductase, azoreductase and ligninolytic enzymes in the biodegradation of azo dyes were mainly reported in several fungal and bacterial strains as mentioned by [67, 71, 83]. Our results are in agreement with [67, 84] who illustrated that laccase and NADH-DCIP reductase increased in the production level by *Bacillus cereus* SKB12 and *G. geotrichum* during biodegradation of textile dyes. However, our results are incompatible with the results of the strain SKB12 which showed a poor production of NADH-DCIP reductase and MnP enzymes. Significantly, none of the tested eight degradative enzymes was detected in the control sample which was not treated in the culture medium with MB dye. These results clearly indicated that all tested degradative enzymes were induced by addition of the synthetic dye to the culture medium. Similarly, a microbial consortium of *Pseudomonas aeuroginosa* BCH and *Providencia* sp. SDS isolated from a soil contaminated with dye showed a potent decolorizing activity of the Red HE3B through a significant increase in the production level of enzymes such as azoreductase, DCIP reductase, veratryl alcohol oxidase and laccase when compared to the control group [85]. Otherwise, [71] stated that NADH-dichloroindophenol oxidoreductase, laccase, LiP, MnP and monooxygenase were the key enzymes in the detoxification of azo dye by *C. tropicalis.*

Remarkably, it is urgent to assess the toxicity of degradation by-products in comparison to MB before treatment to guarantee the feasibility of the consortium and also the safety and toxicity-free nature of such metabolites. Toxicity bioassays were evaluated by several means such as toxicity on invertebrates, mutagenicity, genotoxicity, microbial toxicity and ecotoxicity [24]. However, phytotoxicity and cytotoxicity bioassays were selected in the current study, based on economic, time-saving and feasibility features. Extensively, the phytotoxicity assay has been tested to show the validation of utilization MB-treated wastewater in industrial and ferti-irrigation purposes, especially non-edible crops, to overcome water deficiency problem. By surveying several studies, the phytotoxicity approach was examined via germination test of plant seeds. *Phaselus mungo*, *Oryza sativa* [67], *Vigna radiata* [86], *Triticum aestivum* and *Sorghum vulgare* [17, 78] are the most widely plants used for such bioassay. Herein, microalga *C. vulgaris* was recruited to assess the influence of MB and its degradation metabolites on its growth and photosynthetic activity. It is noteworthy that microalgae are commonly used in toxicity bioassay assessments in aquatic pollutants due to their sensitivity, rapid propagation, high surface of exposure, and higher bioaccumulation capacity as revealed by [87]; consequently, it provided a prompt insight into the quality of bioremediated solution.

Notably, [88] declared that some metabolic outcomes from dye degradation process are frequently more toxic than their parent untreated dyes, indicating the mutagenicity responses in *Salmonella* sp. and mammalian systems. Herein, despite the exact identification of the degradation metabolites were not performed, which will be examined in a future study, the current study revealed that degradation by-products had less toxicity than MB. This was confirmed by maintaining 91.8 and 85.9% of chlorophyll-a and chlorophyll-b content, respectively, relative to 36.4 and 30.4% in MB before treatment. On the contrary, the carotenoids content decreased to 30% upon treatment after sharp uplifting to 149% in untreated MB solution. Interestingly, the carotenoids cope and tolerate toxic substances under stress circumstances by their antioxidant activity [89]. However, for the cytotoxicity bioassay, human cell lines Wi-38 and Vero cells were utilized to evaluate toxicity of MB and its degradation by-product. Maintaining the viability of both normal cells to 90.67% and 99.67%, respectively, unveiled the safety and alleviated toxicity of degradation metabolites compared to 22.43% and 41.17%, respectively in the presence of parent MB solution. Such toxicity data reflected the effectiveness of examined yeast-bacterial consortium in MB degradation to a safe limit.

However, our main target is designing a microbial formula that could assist in azo dye bioremediation in the field or *in-situ* via bioaugmentation process. To achieve this aim in an economic way, the microbial consortium in different formulas were applied in artificially MB-polluted municipal wastewater and industrial effluents, including consortium suspended in optimized slurry (COS) and in distilled water (CDW) either in immobilized and free-suspended states. As observed, the wastewater quality criteria mainly affected the bioremediation rate. That was clearly manifested by the complete removal of 200 mg/L of MB within 84 and 108 h for artificially polluted municipal wastewater by COS of immobilized and free-suspension systems, respectively. On the contrary, 57.39 and 78.46% were eliminated within 144 h via COS of immobilized and free-suspension systems from artificially polluted industrial effluent, respectively. The collective presence of considerable concentrations of phenolic compounds, several heavy metals and oil inhibited relatively examined consortium and retarded its activity. However, the enrichment of municipal effluent with microbial count, nitrogen, sulfate, phosphate contents accelerated the biodegradation process and enhanced the removal rate by this imported consortium.

Remarkably, the entrapment in alginate microspheres exhibited higher efficacy in biodegradation process than freely-suspended state. As highlighted by [90, 91], the superior properties provided by immobilization, including better mechanical strength, stability, tolerance against adverse environmental conditions and hazardous by-products, protection of cells, viability preservation over a prolonged period and simplicity/ easiness of reuse/ recovery could explain such results. In addition, natural/ non-synthetic origin of alginate hydrogel contributed in successful degradation process by its low toxicity and biocompatibility traits. Therefore, entrapment in alginate matrix was frequently employed in biotechnological, medical and environmental applications [92]. Several researches addressed a successful bioremediation of azo dyes by microbial immobilization technique [93–96]. On the other hand, the employment of consortium in optimized slurry (COP) showed improved degradation in both freely-suspended and immobilized compared to that suspended in distilled water (CDW), reflecting the significance of nutrient supplementation to manipulate pollutants. In this context, [60], referred to complex relationship between pollutant removal and nutrient availability which both influenced on microbial performance for bioremediation. Herein, an economically optimized slurry has proven its effectiveness in enhancing the detoxification of MB, enabled the examined consortium to adapt different effluent stressors efficiently. Consequently, it is suggested to successfully transfer the treatment technology to the field such as constructed wetlands.

Despite the presence of a considerable count of native microbes in both examined discharges, their capability to degrade MB was restricted in the range of 5.4–9.2%. Nevertheless, man-made bioremediation technology, viz bioaugmentation, confirmed its potent performance by the range of 42.39–69.38% and 57.39–100% for free and immobilized systems, correspondingly. These results demonstrated the synergistic interaction between the introduced mixed-culture consortium and the indigenous microbiota. Besides, the capability of our examined consortium to withstand a competitive inhibition with native residents, although numerous differences in their metabolic activity owing to their original background, and also tolerate various contents of both effluents, which could represent stressors (e.g., heavy metals, osmotic stress, phenols, etc.) influenced negatively on degradative ability. Interestingly, the stability in degradation activity, perseverance, competitive interaction of imported consortium with native microbiota in a polluted area are considered the decisive keys in determining the result of bioaugmentation, namely success or failure [91, 97]. As mentioned by [98, 99], several studies recorded the unsuccessfulness of employing bioaugmentation in the field or in situ. Additionally, longer treatment time permitted enhanced removal percentage which is in agreement with [99, 100]. Finally, the promising results of MB-detoxifying mixed co-cultures would be invested in studying the dynamics of bioenhancement in various discharge systems, to conform environmental legislations and the limits of its acceptance.

## Conclusions

This study developed promising mixed cultures of yeast (*Rhodotorula* sp.) and bacteria (*Raoultella planticola*; and *Staphylococcus xylosus*) that efficiently degrade MB in a complementary interaction through utilizing molasses as co-substrate. By optimizing nutritional parameters, via statistical approach such as PBD and CCD, maximum remediation of MB (200 mg/L) was achieved within 72 h of incubation by a threefold enhancement relative to basal conditions. The mechanistic pathway for MB degradation was concluded from the assays of intracellular activity of NADH-reductase, DCIP-reductase, azoreductase, laccase, nitrate reductase, LiP, MnP, and tyrosinase, which indicated the maximum expression of enzymes recorded after 72 h of incubation. Monitoring UV–Vis spectral and FTIR analysis of degradation metabolites reflected the breakdown of MB entity. However, the toxicity results reflected the non-toxic nature of degradation by-products in both phytotoxicity and cytotoxicity levels, reflecting the efficiency of designed consortium in effective detoxification of MB. Furthermore, by applying such co-cultural system, freely-suspended and immobilized states, in treatment of MB-artificially contaminated real effluents, via bioaugmentation technology, enhanced remediation is observed. The results revealed a successful degradation that reached up to 100% MB-elimination in domestic wastewater by 84 and 108 h, respectively for immobilized and feely-suspended states, whereas 57.39 and 78.46% were removed by 144 h through the activity of free-suspension and immobilized systems in industrial effluent, respectively, reflecting the adaptation of consortium with native inhabitant. Subsequently, this constructed consortium has the potential to be recruited in treatment of azo dye from effluents with different physicochemical contents.
